# Comparative Analysis of Clinical Outcomes and Financial Aspects of Phototherapies and Immunotherapy for Cancer

**DOI:** 10.1002/advs.202417657

**Published:** 2025-06-19

**Authors:** Deepak S. Chauhan, Kun Shao, Roopa Hebbandi Nanjundappa, Daniel‐Jiajun Yu, Marianne Stanford, Huile Gao, Channakeshava Sokke Umeshappa

**Affiliations:** ^1^ Department of Microbiology and Immunology Dalhousie University Halifax NS B3H 4R2 Canada; ^2^ Department of Pediatrics IWK Research Center Halifax NS B3K 6R8 Canada; ^3^ State Key Laboratory of Fine Chemicals Dalian University of Technology Dalian 116023 China; ^4^ Faculty of Pharmacy Universite de Montreal Montreal QC H3T 1J4 Canada; ^5^ Key Laboratory of Drug‐Targeting and Drug Delivery System West China School of Pharmacy Sichuan University Chengdu 610041 China

**Keywords:** clinical trials, immunotherapy, photodynamic therapy, photoimmunotherapy, photothermal therapy

## Abstract

Photo‐responsive/activable nanomedicine‐driven therapies have emerged as a transformative approach to effectively target and eliminate tumors across different types of solid cancers. When synergistically combined with immunomodulators, these therapies significantly enhance anti‐cancer efficacy. This comprehensive review examines the current advancements in photo‐responsive/activable immunomodulator‐loaded nanomedicines (PINs), providing a detailed analysis of their preclinical development, clinical translation, and associated research funding. The diverse PINs are systematically categorized, innovative design strategies are explored, and the underlying photo‐responsive/activable and immunomodulatory mechanisms are elucidated. By integrating clinical advancements with technical innovations and financial perspectives, this review bridges the translational gap, underscoring the immense potential of PINs to revolutionize cancer therapy. This comprehensive resource is indispensable for researchers striving to bring phototherapies to clinical fruition in the battle against cancer.

## Graphical abstract

This review compares clinical outcomes, translational progress, and global funding trends across cancer phototherapies—photodynamic, photothermal, and photoimmunotherapy—and conventional immunotherapy. It highlights differences in treatment efficacy, clinical trial status, financial investment, and regulatory challenges, providing a comprehensive overview to guide future development and policy decisions in cancer nanomedicine and combination therapy strategies.

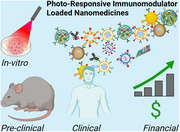

## 1 Introduction

Cancer remains one of the most pressing medical challenges and continues to be the second leading cause of mortality worldwide, necessitating need for innovative treatment strategies.^[^ ^1^ ^]^ Over the last decade, the field of cancer nanomedicine has witnessed significant advancements aimed at improving therapeutic efficacy while minimizing adverse effects. Nanomedicines, including liposomes and antibody‐drug conjugates, have shown great promise in targeted drug delivery, reducing systemic toxicity, and enhancing patient outcomes.^[^ ^2^ ^]^ However, despite these advancements, issues such as nanomedicine stability, residual systemic toxicity, and the complex resistance mechanisms within tumor microenvironments persist. These challenges emphasize the need for integrating nanomedicine with localized physical therapies, such as photodynamic therapy (PDT) and photothermal therapy (PTT), to overcome existing limitations and achieve more effective cancer treatment.^[^ ^3^ ^]^

Recent advancements have introduced transformative therapeutic approaches, such as PDT and PTT, to address the challenges associated with traditional cancer treatments.^[^ ^4^ ^]^ Compared to traditional chemotherapy, PDT and PTT employ near‐infrared (NIR) light‐absorbing nanoparticles and dyes to selectively generate reactive oxygen species (ROS) or heat within tumor cells or the tumor microenvironment upon exposure to light. PDT and PTT's minimally invasive nature offers reduced recovery times, sparing healthy tissues from unwanted side effects seen with conventional treatments. However, considerations regarding the optimal design of PDT and PTT agents, limitations in NIR light penetration, potential long‐term toxicity due to incomplete clearance, and challenges in treating metastatic tumors have driven the evolution of photo‐responsive/activable immunomodulator‐loaded/conjugated nanomedicines (PINs)‐driven photo‐immunotherapies (PIT).^[^ ^5^ ^]^ By merging phototherapies with immunotherapy, PIT presents a multi‐pronged strategy for fighting cancer. The NIR light‐driven photothermal effect not only directly eliminates cancer cells but also stimulates systemic anti‐cancer immune response with the help of immunomodulators, thus aiding in the control of metastasis.^[^ ^4^, ^6^ ^]^ This innovation capitalizes on the synergy between the precision of PDT/PTT and the systemic impact of immunotherapy.

While PIT shows significant promise, its clinical success hinges on effective immune activation and the management of potential immune‐ and nanomedicine‐related adverse events. In this review, we thoroughly examine the progress in PIT research, with a particular emphasis on aspects beyond solely phototherapies like PTT and PDT, and compare these advancements with those in cancer immunotherapy. Furthermore, we discuss the underlying reasons for the slow pace of the translation of phototherapies and draw insights from the current funding trends for clinical trials. By doing so, we illuminate potential pathways for future research endeavors to enhance the translation of PIT from the laboratory bench to the clinical bedside.

**Figure 1:**
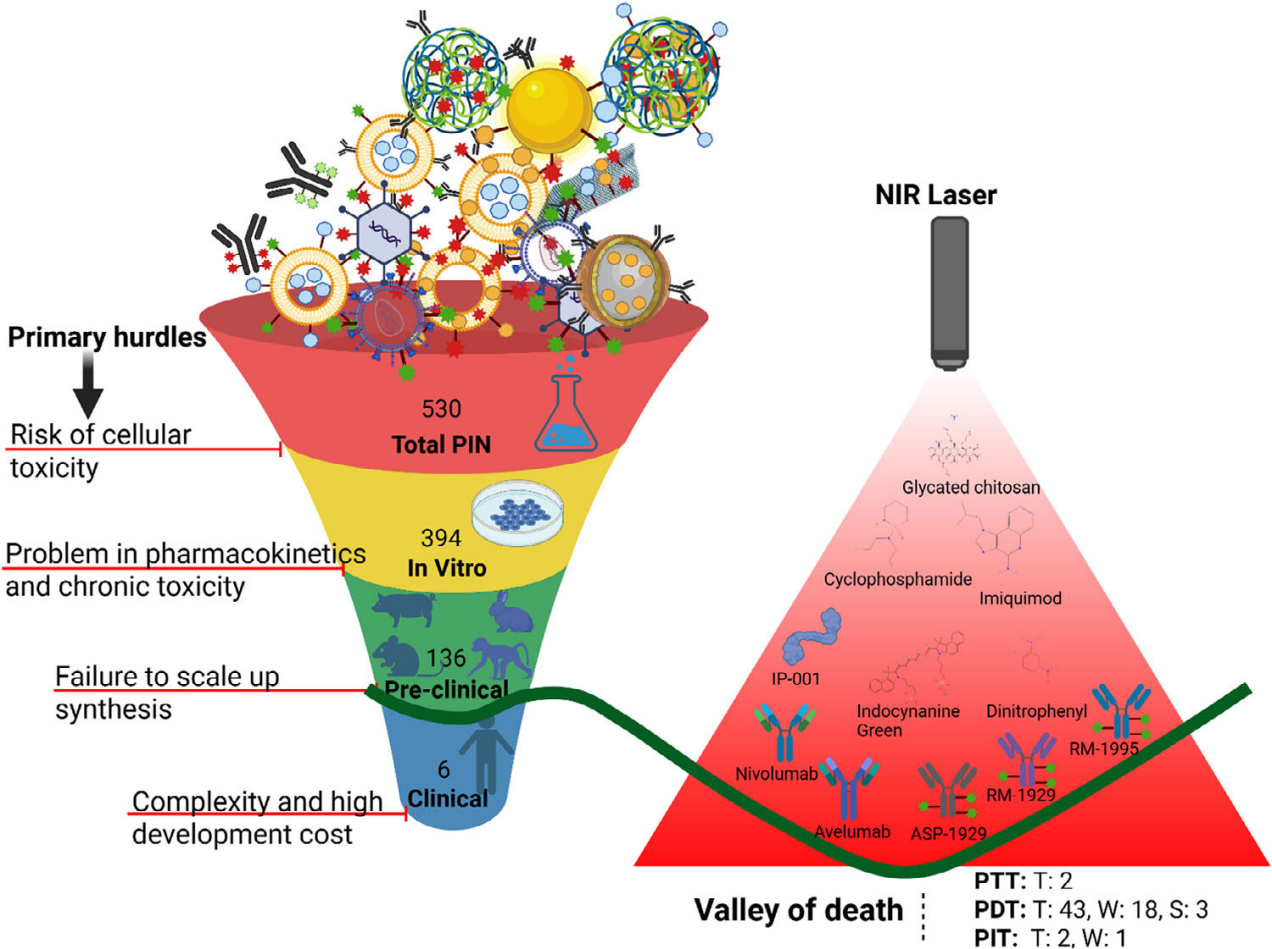
Primary hurdles and valley of death in PIT development: Out of 530 studies analyzed, 394 studies have completed the in vitro validation, 136 completed the preclinical studies in mice, canine, pigs, rabbits, and primates, and 6 studies related to clinical trials have been published. The primary hurdles preventing successful transition from one stage to the next are indicated on the red color stop line. The figure on the right side depicts immunomodulators navigating through the “valley of death” under NIR light. Abbreviations: T: terminated, W: withdrawn, S: suspended clinical trials. Search engine: PubMed.

Based on our comprehensive analysis using different combinations of keywords (photoimmunotherapy, mice, canine, pigs, rabbits, primates, and clinical trials) and logic gates of research articles indexed in PubMed, NIH Reporter, and ClinicalTrials.gov, we identified a total of 530 original research articles focused on PIT till 6th April 2025, **Figure** **1**. Of these, 394 studies have completed in vitro investigations, while 136 studies have advanced to preclinical evaluations in animal models. Among the 14 clinical trials, 6 clinical studies have been published. This disparity highlights the “valley of death,” a critical phase in drug development where many promising therapies fail to progress from laboratory success to clinical application due to challenges like scalability, regulatory hurdles, and unforeseen side effects in humans.

## 2 Types, Design Strategy, and Mechanisms of PINs

**Figure 2:**
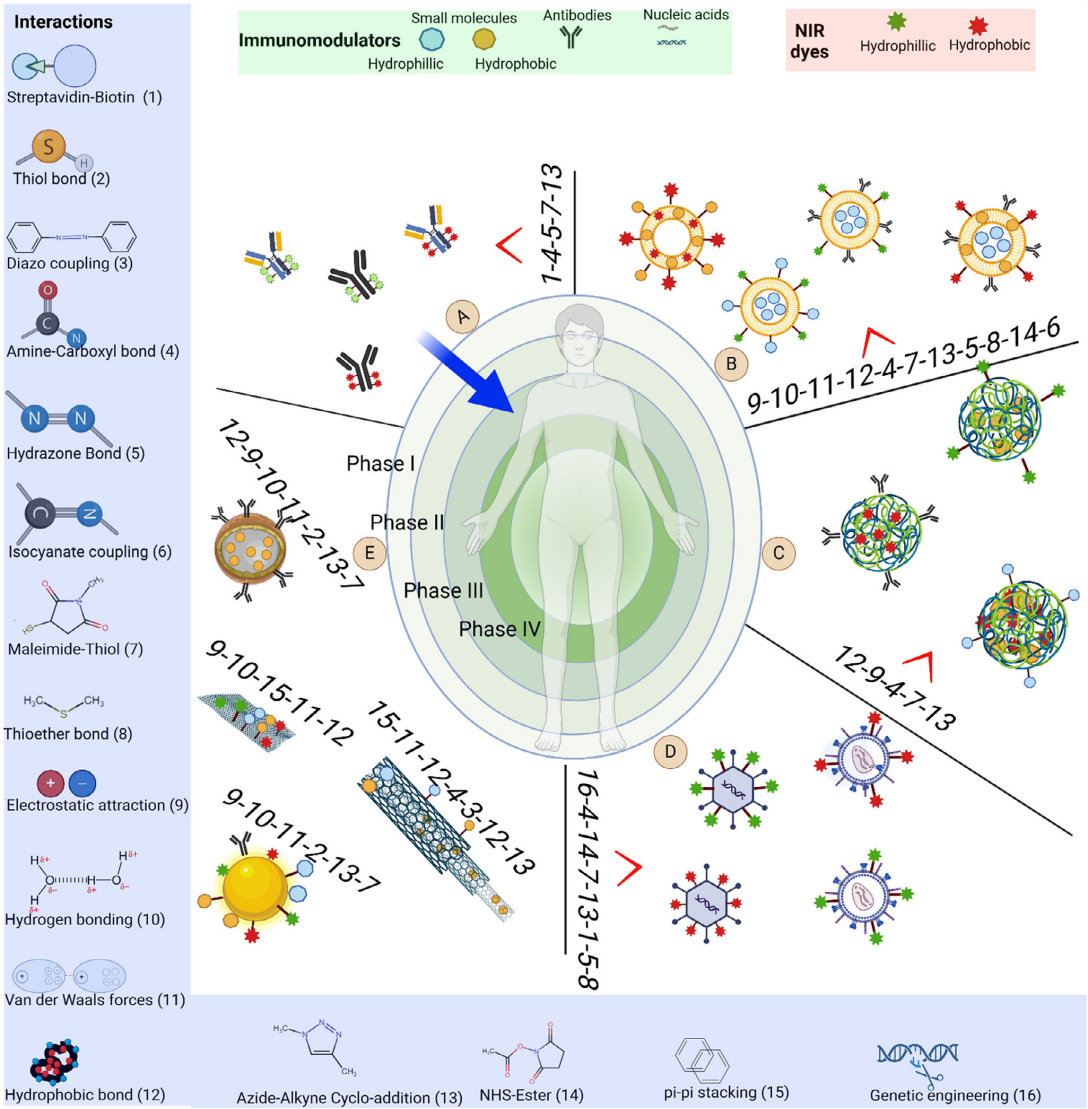
Different categories and types of interactions in PINs. A) Antibody‐dye conjugate, B) Liposome‐based PINs. C) Polymeric nanoparticle‐based PINs, D) Oncolytic virus‐based PINs, E) Inorganic–organic hybrid nanomaterials‐based PINs. A blue solid arrow indicates the transition of antibody‐based PINs from preclinical to clinical trials (Phase III). The numbers on each PIN type indicate the potential bonding for conjugating/loading NIR dye or immunomodulator to the nanocarriers. The sequence of numbers follows a hierarchical ranking, where the first number represents the most commonly used bonding type, followed by less frequent interactions (see interactions).

PINs can be classified into several different categories based on the composition and structure, as shown in **Figure** **2**A–D. The first category (A) of PINs is consist of antibody‐dye conjugate, **Table** **1**.^[^ ^7^ ^]^ The second (B) and third (C) categories are photo‐responsive/activable polymeric^[^ ^8^ ^]^ and lipid^[^ ^9^ ^]^ nanoparticles loaded with immunomodulators. The fourth (D) category involves engineering immunomodulating viruses for NIR light response.^[^ ^10^ ^]^ The last and fifth (E) category is combinatorial in nature, wherein materials are combined from the above categories with inorganics materials like gold^[^ ^11^ ^]^ carbon nanotubes,^[^ ^12^ ^]^ and graphene,^[^ ^13^ ^]^ to improve therapeutic efficacy. Additionally, biohybrid PINs have been developed by coating the particle with the cell membranes of different immune cells to develop biomimetic, trigger‐responsive, targeted PINs.^[^ ^14^ ^]^ The biomimetic PINs help in the specificity of PINs to the tumor location by increasing the blood circulation time as well as targeting ligands, which are further controlled by the external stimulus of light. However, each category of PINs presents unique challenges.^[^ ^15^ ^]^ For example, antibody‐dye conjugates face issues with specificity, stability, and production costs.^[^ ^16^ ^]^ Polymeric and lipid nanoparticles struggle with biocompatibility, controlled release, and scalability.^[^ ^17^ ^]^ Engineering immunomodulating viruses for NIR light response raises safety concerns, regulatory hurdles, and production difficulties.^[^ ^18^ ^]^ Combinatorial PINs, incorporating inorganic materials like gold, carbon nanotubes, and graphene, pose toxicity risks and challenges in clearance, biodistribution, and manufacturing. Biohybrid PINs, while designed to improve specificity and reduce immune response, encounter issues with immune recognition, complex production, and maintaining stability and functionality.^[^ ^19^ ^]^ Addressing these challenges is crucial for advancing the clinical application of PINs.

**Table 1:** Representative of antibody‐based PINs developed for preclinical studies.

Year	Immunomodulant	Photo‐responsive/activable agent	Wavelength/power density (time)	Target indication	Animal model	Refs.
1972	Trastuzumab	Irdye‐700DX	685–695 nm at 50 and 100 J cm^−2^	Lung cancer	Calu3 tumor‐bearing mice	[20]
1983	Mab	Hematoporphyrin	Incandescent light for 4 h	Myosarcoma M‐1	M‐1 tumor‐bearing DBA/2J mice	[21]
1994	Mab OC125	CD‐Ce6	656 nm at 15 J cm^−2^	Ovarian cancer	NIH:OVCAR3 tumor‐bearing mice	[22]
2005	Mab FSP 77, 17.1A, 35A7	Porphyrin isothiocyanates	630 nm at 10 J cm^−2^	Colon cancer	LS174T and SKOV3 tumors bearing mice	[23]
2011	Trastuzumab and panitumumab	IR‐700	670–690 nm at 2.6 mW cm^−2^	Epidermoid carcinoma	A431 and 3T3 tumors bearing mice	[24]
2012	Panitumumab	IR‐700	690 nm at 50 J cm^−2^	Epidermoid carcinoma	A431 tumor‐bearing mice overexpressing HER1	[25]
2013	Panitumumab	IR‐700	690 nm at 50 J cm^−2^	Epidermoid carcinoma	A431 tumor‐bearing mice	[26]
2014	Cetuximab and panitumumab	IR‐700	670–710 nm at 50 and 100 J cm^−2^	Epidermoid carcinoma, and breast cancer	A431 and MDAMB468 tumors bearing mice	[27]
2017	FAP‐specific scfv	ZnF16Pc Loaded Ferritin	671 nm at 300 mW cm^−2^	Breast cancer	4T1 tumor‐bearing mice	[28]
2018	Trastuzuma, panitumumab, and cetuximab	IR‐700	690 nm at 16 J cm^−2^	Epidermoid carcinoma and adenocarcinoma	A431 and MDAMB468 tumors bearing mice	[6]
2019	Indoleamine 2,3‐dioxygenase	Liposome based porphyrin–phospholipid conjugate	660 nm at 400 mW cm^−2^ for 10 min	Breast cancer	4T1 tumor‐bearing mice	[9]
2022	PD‐L1 aptamer and OXA	Zr_6_ MOF (PCN‐224)	640 nm at 100 mW cm^−2^ for 30 min	Colorectal cancer	MC38 bilateral tumor‐bearing mice	[29]
2022	Cetuximab	Irdye‐700DX	100–150 J cm^−2^ of 690 nm light at 150 mW cm^−2^	Colorectal cancer and fibrosarcoma	CT26 and MCA205 tumor‐bearing mice	[30]
2023	*α*‐PD‐L1 antibody	Au Pd hss	808 nm at 500 mW cm^−2^ for 10 min	Breast cancer	4T1 tumor‐bearing mice	[31]
2023	Panitumumab	Irdye‐700DX	690 ± 5 nm at 30 J cm^−2^	Adenocarcinoma	MDAMB468 bone metastases mice	[32]
2024	HER2‐targeting peptide	Pyropheophorbide	660 nm at 90 J cm^−2^ for 6 min	Breast cancer	TUBO tumor‐bearing mice	[33]
2025	*α*‐PD‐L1 antibody	Gadolinium‐doped carbon dots	650 nm at 500 mW cm^−2^ for 5 min	Melanoma and breast cancer	B16F10 and 4T1 tumor‐bearing mice	[34]

Abbreviations: Mab: monoclonal antibodies, FAP: fibroblast activation protein, scfv: single chain variable fragment, FSP: fibroblast specific protein, CD‐Ce6: chlorin e6‐conjugated β‐cyclodextrin, Zr6 MOF: metal‐organic framework nanoparticle porphyrinic (PCN‐224) core, Au Pd hss: gold‐palladium heterostructures, OXA: oxaliplatin, HER2: human epidermal growth factor receptor 2.

The design of PINs for maximum therapeutic efficacy with minimum side effects requires multidisciplinary expertise. It involves the selection of immunomodulators, carriers, and considerations of pharmacokinetic behavior, distribution, and metabolism. Below, we have discussed the principal factors for the successful development of PINs.

### 2.1 Size, Shape, and Surface of PINs

The size of PINs is important for generating an efficient immunomodulatory effect; for example, the size of immune cells is different in different species.^[^ ^35^ ^]^ Thus, the size of PINs should be tuned for the optimized uptake by the human immune cells during the translational studies. Most of the PINs are designed in the order of 10–1000 nm, which is multi‐order smaller than the immune cells.^[^ ^36^ ^]^ This size range aids in lymphatic drainage and cellular internalization. While larger PINs have a high chance of entrapment in the extracellular matrix and, thus, preventing interaction with immune cells.^[^ ^37^ ^]^ Different immune cells show different preferences for the size; macrophages can easily engulf 2–3 µm size microparticles, while other immune cells prefer nano PINs over micro‐sized PINs.^[^ ^39^ ^]^

**Figure 3:**
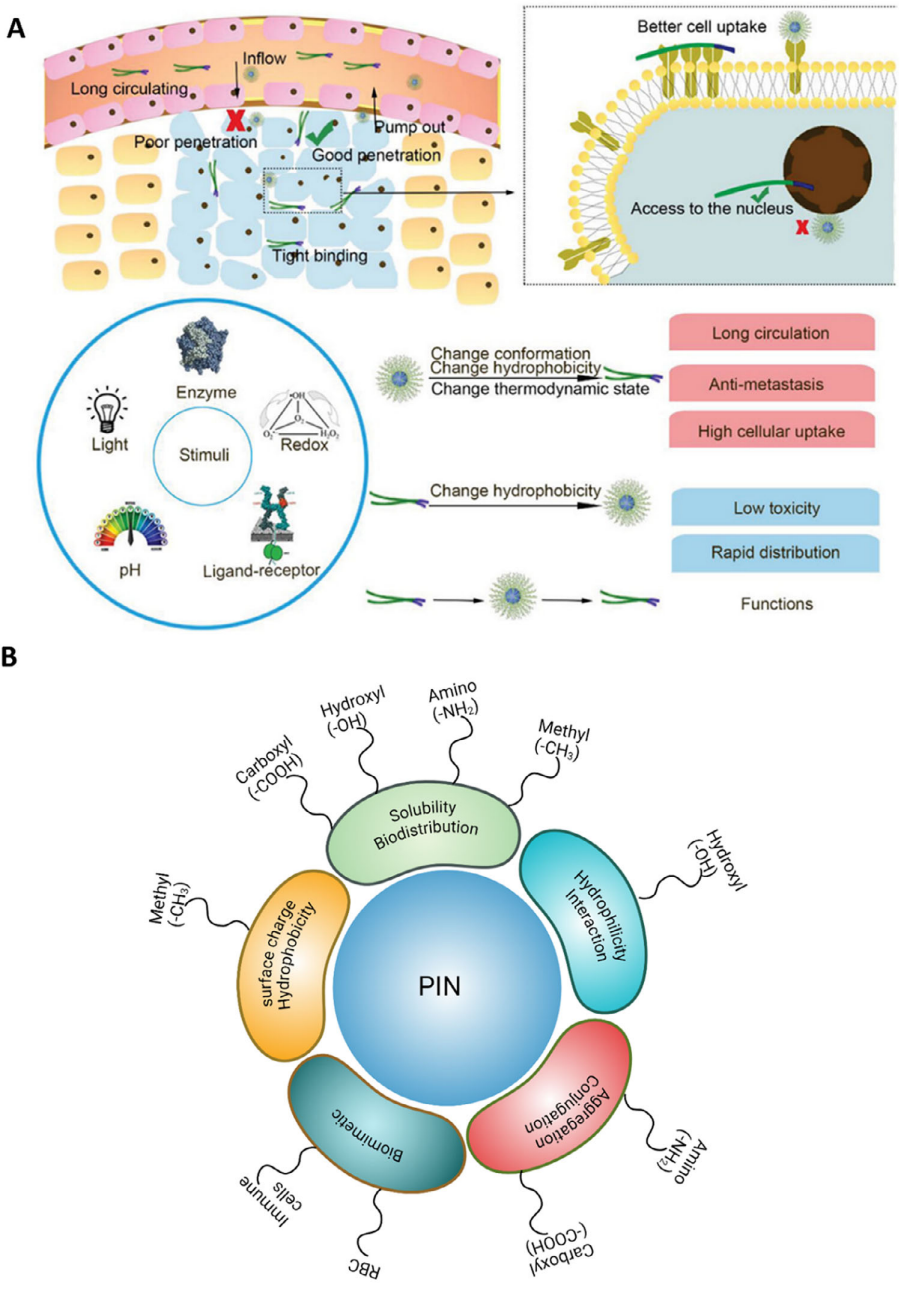
Influence of external stimuli and surface modifications on nanoparticle behavior and therapeutic efficacy. A) A concise depiction of how nanoparticle penetration, uptake, and stimuli‐responsive behavior influence delivery effectiveness. Light stimuli primarily exert effects via heat (photothermal response), enhancing permeability and cellular uptake. Other external stimuli, including enzymes, redox conditions, pH, and ligand–receptor interactions, can trigger conformational or hydrophobicity changes, improving circulation, tumor penetration, and targeted drug release. B) Surface functionalization of PINs and their role in biodistribution and targeting. Surface modifications such as carboxylation, hydroxylation, amination, and methylation influence solubility, biodistribution, cellular interactions, and immune targeting. Additionally, biomimetic coatings derived from immune cells or RBCs enhance tumor targeting and prolong circulation time. Adapted with permission^[^ ^38^ ^]^ Copyright2011, Wiley.

The shape of PINs is a critical factor influencing their interactions with immune cells and biodistribution. While spherical PINs are widely regarded as the gold standard due to their extensively studied mechanisms of endocytosis and cellular uptake,^[^ ^40^ ^]^ other morphologies, such as rods, ellipticals, stars, and needles, induce distinct immune responses. For example, rod‐shaped PINs exhibit prolonged circulation time and enhanced antigen presentation, making them favorable for immune activation.^[^ ^41^ ^]^ In contrast, platelet‐like or star‐shaped structures can modulate macrophage polarization, promoting a pro‐inflammatory response, which is beneficial for tumor suppression.^[^ ^42^ ^]^ Additionally, needle‐like PINs may demonstrate higher cellular adhesion and uptake efficiency, influencing their accumulation in lymphoid organs and antigen‐presenting cells (APCs).^[^ ^43^ ^]^ These shape‐dependent effects play a crucial role in determining PIN‐mediated immune modulation and biodistribution, **Figure** **3**A.^[^ ^38^, ^44^ ^]^

The surface modifications of PINs, such as carboxylation, hydroxylation, amination, and methylation, not only enhance immunomodulation, biodistribution, and biocompatibility but also play a crucial role in active targeting and transcytosis, facilitating more precise delivery to tumor sites, Figure 3B.^[^ ^45^ ^]^ For example, carbon nanotubes functionalized with carboxyl and amine groups, along with acid treatment, enhances their immunomodulatory responses on monocytes by perturbing the signaling pathways of IL‐6, TNF‐alpha, CD40, and dendritic cells (DCs) maturation, as well as improving aqueous solubility.^[^ ^46^ ^]^ These surface modifications also offer unique opportunities to tailor the interaction of PINs with biological systems, thereby influencing their biodistribution profiles and, ultimately, their therapeutic efficacy. For instance, Carboxylation on the surface of PINs can enhance the dispersibility and solubility of PINs in aqueous environments.^[^ ^47^ ^]^ This modification may improve biodistribution by preventing aggregation and facilitating a more homogeneous distribution within the bloodstream.

Additionally, carboxyl groups can serve as functional handles for further conjugation with targeting ligands,^[^ ^48^ ^]^ allowing for enhanced recognition of specific receptors on target cells, which can direct PINs to tumor sites with greater precision. The hydroxylation and amination on the surface of PINs play crucial roles in shaping the biodistribution of PINs; for example, hydroxyl groups can impart increased hydrophilicity to the surface of PINs, potentially promoting their circulation in the bloodstream and facilitating interactions with blood components.^[^ ^49^ ^]^ Similarly, amination can introduce amino groups that enable electrostatic interactions with proteins and cell membranes, influencing the uptake and distribution of PINs in various tissues.^[^ ^50^ ^]^ Moreover, methylation of PINs can alter the surface charge and hydrophobicity of PINs, affecting their interactions with biological barriers such as cell membranes and the extracellular matrix.^[^ ^51^ ^]^ This modification may also influence the rate of clearance from circulation, the extent of tissue penetration, and the overall distribution pattern of PINs within the body.

**Figure 4:**
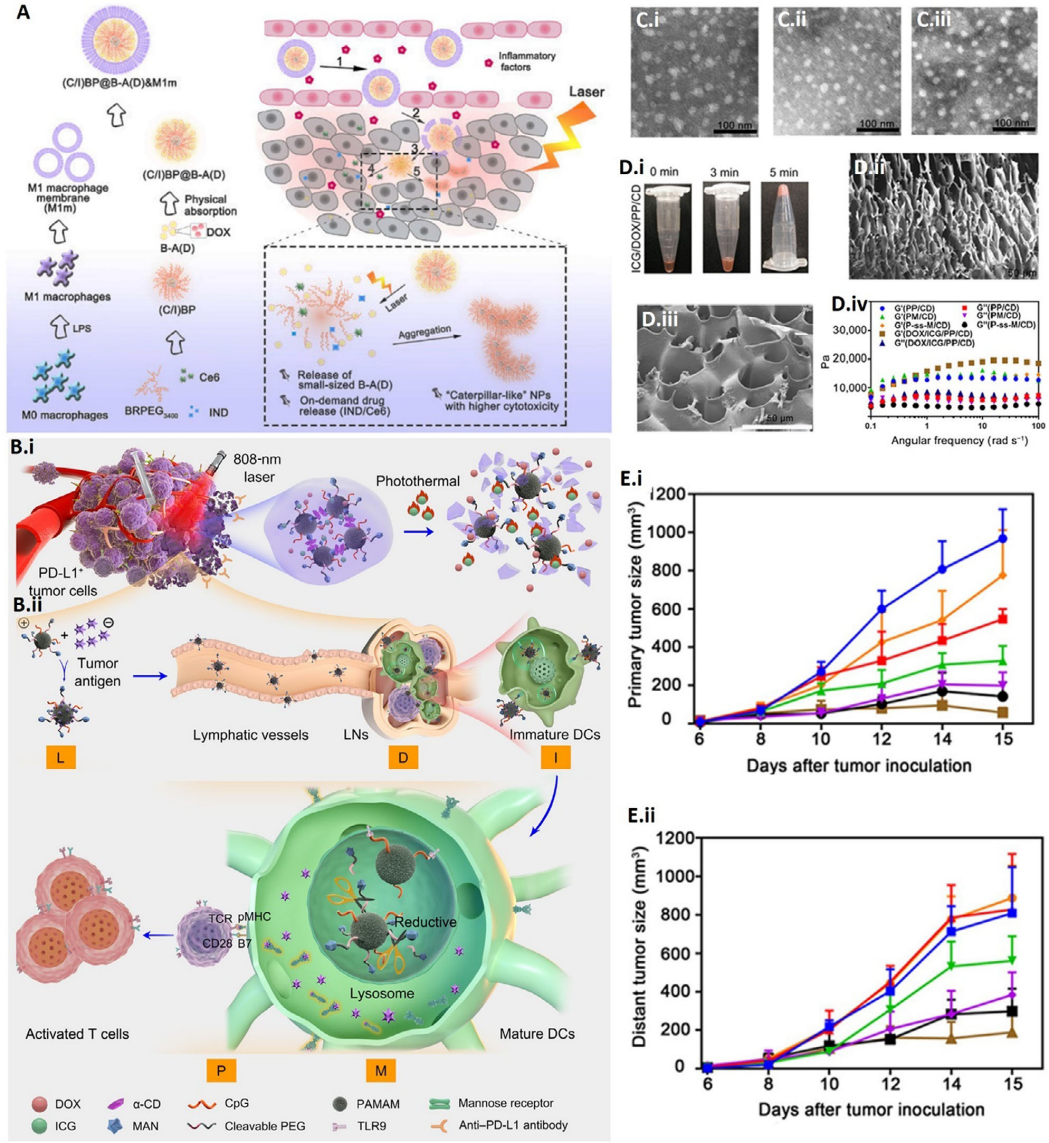
Combinatorial photoimmunotherapeutic approach for cancer treatment. A) Schematic showing the synthesis of macrophage membrane‐coated PIN and laser‐triggered release of an immunomodulator (IND), a photosensitizer (Ce6), and cytotoxic nanoparticles. B‐i, ii) Nanogel for the photoimmunotherapy of cancer. The assembly of the combined treatment plan and the release mechanism of CpG‐P‐ss‐M (i). A simplified explanation of CpG‐P‐ss‐M‐induced DCs maturation for cancer immunotherapy (ii). The letters LDIMP, highlighted in orange, symbolize the loading of tumor‐specific antigens through drug delivery systems (DDS), their transport to lymph nodes (LNs), internalization by DCs, maturation of DCs leading to the expression of costimulatory molecules, and the presentation of peptide‐MHC‐I complexes to T cells, respectively. C‐i–iii) TEM images of CpG‐PP (i), CpG‐PM (ii), and CpG‐P‐ss‐M (iii). D‐i–iv) Photograph showing the gelation process following ultrasonication (i), scanning electron microscopy images of PP/CD gel (ii, iii), rheological properties of gels as a function of frequency (iv). E‐i, ii) Tumor growth curves for primary (i) and distant (ii) tumors (*n* = 6). Reproduced with permission.^[^ ^4f,g^ ^]^ Copyright 2020, Science; Copyright 2020, Elsevier. Abbreviations: M1m: M1 macrophage membrane, DOX: doxorubicin, BRPEG3400: PEGylated bilirubin, LPS: lipopolysaccharide, Ce6: chlorin e6, IND: indoximode, B‐A(D): DOX inserted and bovine serum albumin‐protected gold nanoclusters (B–AuNCs), (C/I)BP: PEGylated bilirubin to co‐deliver hydrophobic Ce6 and IND, (C/I)BP@B‐A(D): physically adsorbed (C/I)BP on B‐A(D), (C/I)BP@B‐A(D)&M1m: macrophage membrane coated (C/I)BP@B‐A(D), α‐CD: α‐cyclodextrin, MAN: reductive‐cleavable 4‐aminophenyl‐α‐d‐mannopyranoside, PAMAM: polyamidoamine dendrimer, pMHC: Peptide major histocompatibility complex.

To enhance uptake, the surface of PINs is also modified with ligands that selectively recognize the overexpressed receptors on immune cells. Following this strategy, we have also modified the surface of PINs with the biological components (membrane or vesicles) derived from the immune cells, known as biomimetic PINs, to ensure efficient uptake by tumors and increase circulation time, **Figure** **4**A.^[^ ^4g^ ^]^ These PINs, when injected into the body, behave like pseudo‐immune cells, allowing them to bypass biological barriers through transcytosis mechanisms and efficiently penetrate tumor tissues, increasing retention time and reducing off‐target effects. This biomimetic strategy improves systemic circulation, ensuring deep tumor infiltration and enhanced therapeutic efficacy. The induction of immunogenic cell death (ICD) by PIT‐driven combinatorial treatment resulted in the suppression of primary tumor growth in both the 4T1 and B16F10 mouse tumor models, with no observed adverse effects. It also prevented primary tumor recurrence and metastasis.

The incorporation of these surface modifications not only optimizes biodistribution profiles but also enhances active targeting, transcytosis, and immune modulation, leading to improved tumor localization and superior clinical outcomes in photoimmunotherapy‐based cancer treatment. Understanding the impact of each modification is essential for the rational design of personalized PIN‐based immunotherapies.

### 2.2 Immunomodulant for PIN

The immunomodulant is a small molecule, material, or biological entities with immunosuppressive or immunostimulatory properties to restore the normal function of immune cells.^[^ ^52^ ^]^ A wide range of immunomodulants have been used in PIT, including checkpoint inhibitors,^[^ ^53^ ^]^ vaccines,^[^ ^54^ ^]^ oncolytic viruses (OV),^[^ ^55^ ^]^ cytokines,^[^ ^56^ ^]^ toll‐like receptors (TLRs) agonist,^[^ ^57^ ^]^ adoptive T cell therapies (ACT)^[^ ^58^ ^]^ and small molecules.^[^ ^59^ ^]^ Besides this, several NIR light‐responsive biomaterials, such as gold nanoshells,^[^ ^60^ ^]^ nanorods,^[^ ^61^ ^]^ carbon nanotubes,^[^ ^62^ ^]^ and graphene^[^ ^63^ ^]^ also exhibit immunomodulatory properties after surface functionalization. The immunomodulants have been used alone or in combination with anticancer drugs to achieve the desired therapeutic efficacy in PIT.^[^ ^64^ ^]^ Below, we have discussed some commonly used immunomodulators.

#### 2.2.1 Checkpoint Inhibitors

Checkpoint inhibitors, such as anti‐ programmed cell death protein 1 (anti‐PD‐1) and anti‐cytotoxic T‐lymphocyte‐associated protein 4 (CTLA‐4), are being investigated in the context of PIT to augment the immune response against tumors while sensitizing cancer cells to light‐induced cytotoxicity.^[^ ^53^ ^]^ In addition to these checkpoint inhibitors, other checkpoint inhibitors and antibodies are also being explored for their potential synergistic effects,^[^ ^65^ ^]^ Table 1. We have also shown that combining checkpoint inhibitors with PIT can synergistically overcome the immunosuppressive tumor microenvironment, boost anti‐tumor cell‐mediated immunity, and facilitate enhanced tumor cell death,^[^ ^4f^ ^]^ Figure 4B–E. Mechanistically, these inhibitors block immune checkpoints, reviving the immune system's recognition and attack on cancer cells. When used with PIT, this combination activates immune responses and potentiates the effects of phototherapies, making cancer cells more vulnerable to destruction upon exposure to specific wavelengths of light. This strategy is particularly important in cancers where immune suppression is prominent, as blocking checkpoints such as PD‐1 or CTLA‐4 reactivates the immune surveillance that tumors often evade.

The mechanistic interactions in the context of PIT become more intricate when these inhibitors are introduced. PIT, which uses targeted light‐based therapies to selectively damage tumor cells, can further enhance the immune response initiated by checkpoint inhibitors. For example, anti‐PD‐1 antibodies, as explored in clinical trials such as NCT03277638, work by inhibiting the interaction between PD‐1 on T‐cells and PD‐L1 on tumor cells, thereby preventing the “off” signal that cancer cells use to evade immune attack. When combined with PIT, this blockage allows T‐cells to remain activated, and the light‐triggered cytotoxicity of PIT amplifies the overall anti‐tumor effect by increasing tumor antigen release.^[^ ^30^, ^66^ ^]^ This release enhances antigen presentation, thereby driving more T‐cell recruitment and activation, creating a feedback loop that strengthens the immune response against the tumor.

Moreover, anti‐CTLA‐4 antibodies, which function by inhibiting the early stages of T‐cell activation, have been shown to complement PIT by enhancing the immune system's ability to mount a sustained response to tumor antigens exposed during the light‐induced destruction of tumor cells.^[^ ^67^ ^]^ For instance, in preclinical models combining anti‐CTLA‐4 with PIT, tumor regression was more profound, as the therapy not only increased immune cell infiltration into the tumor but also helped overcome the immunosuppressive microenvironment typically found in solid tumors.^[^ ^67^, ^68^ ^]^ This synergistic effect is crucial in cancer types that are resistant to immune checkpoint inhibition alone.

Clinical trials such as NCT03277638 and NCT05669352 also explore critical factors like treatment sequencing, dosing, and patient selection criteria to optimize this combination. Determining the optimal timing of checkpoint inhibitor administration relative to PIT could significantly enhance therapeutic efficacy. Administering checkpoint inhibitors before PIT could prime the immune system to be more responsive to the tumor antigens released by phototherapy. Alternatively, post‐PIT administration could amplify the immune response to the damage caused by light therapy.

Despite promising preclinical and early clinical results, the intricate interactions between checkpoint inhibitors and PIT demand further exploration. One challenge lies in fully elucidating how these therapies modulate the tumor microenvironment. For example, PIT may increase the immunogenicity of tumor cells by enhancing dendritic cell activation and promoting the infiltration of cytotoxic T‐cells, but it may also cause the release of immunosuppressive factors, such as regulatory T‐cells (Tregs) or myeloid‐derived suppressor cells (MDSCs). This duality underscores the importance of optimizing combination therapies to prevent potential tumor relapse. Further studies are needed to refine these therapeutic strategies, focusing on biomarkers that predict responsiveness to the combination of PIT and checkpoint inhibitors, the role of the tumor microenvironment in modulating treatment response, and the best strategies to limit toxicity while maximizing therapeutic benefits.

#### 2.2.2 Vaccines

The combination of vaccines with PINs leverages the ability of PINs to act as in situ antigen depots, enhancing the immune system's capacity to recognize and target tumor‐specific antigens. Vaccines in cancer immunotherapy are designed to prime the immune system to recognize tumor‐specific antigens, stimulating a targeted immune response against cancer cells.^[^ ^54^ ^]^ Simultaneously, PIT employs light‐sensitive compounds to selectively destroy cancer cells upon light activation. However, the direct application and optimization of vaccines within PIT protocols remain largely investigational. The challenges include identifying appropriate tumor‐specific antigens, designing effective vaccines, and determining the most advantageous combination strategies with phototherapies. Toward this, we have also developed combinatorial PINs to form in situ vaccines that can generate tumor‐specific antigen depots and thus anti‐tumor immunity,^[^ ^4f,g^ ^]^ Figure 4B–E. The advancements in personalized medicine and immunotherapeutic approaches may pave the way for more tailored vaccine strategies integrated into PIT, but specific methodologies and clinical outcomes in this intersection will require further research and clinical trials for validation and optimization.

#### 2.2.3 Oncolytic Viruses

The combination of OV with PIT holds promise in cancer treatment due to their complementary mechanisms in targeting cancer cells and eliciting immune responses.^[^ ^55^ ^]^ OV, such as modified herpes simplex virus, adenovirus, or vesicular stomatitis virus, are engineered to selectively infect and replicate within cancer cells, causing their destruction.^[^ ^69^ ^]^ In PIN‐mediated combinatorial therapy, photo‐responsive/activable agents, such as photosensitizers and photothermal agents, induce cell death in caner cells upon exposure to specific wavelengths of light. Simultaneously, these viruses also stimulate immune response against the tumor. Studies have explored combining OVs with PIT to amplify their respective anticancer effects. For example, researchers have investigated administering OVs first to prime the tumor microenvironment, making cancer cells more susceptible to subsequent PIT.^[^ ^70^ ^]^ Alternatively, employing PIT before OVs might sensitize cancer cells to viral infection and enhance viral replication within the tumor.

Preclinical research using various OVs in combination with PIT agents has shown encouraging results in animal models.^[^ ^70^, ^71^ ^]^ These studies demonstrate improved killing of tumor cells, enhanced antitumor immune responses, and prolonged survival compared to monotherapies. However, translating these findings to clinical applications requires further investigation. The optimal dosing, timing, and genomic sequences of OV and PIT administration need refinement. The ongoing research endeavors aim to elucidate the complex interactions between OVs and PIT and optimize protocols for improved therapeutic outcomes in cancer patients.^[^ ^72^ ^]^

#### 2.2.4 Cytokines

Cytokines, such as interleukins (IL) and interferons (IFNs), can modulate immune responses and promote antitumor immunity.^[^ ^56^ ^]^ In the context of PIT, these cytokines might be employed to potentiate the immune response triggered by light‐induced cancer cell death. Preclinical studies have investigated using cytokines in conjunction with PIT to amplify the immune response against cancer cells. For instance, researchers have explored delivering cytokines directly into tumors or systemically alongside PIT to activate immune cells, enhance their infiltration into the tumor microenvironment, and stimulate an antitumor immune response.^[^ ^73^ ^]^ IL‐2, known for its role in stimulating T cells and natural killer (NK) cells, has been used in combination with PIT to treat cancer in animal models.^[^ ^56^ ^]^ This approach potentiated the anti‐tumor immunity, facilitating the recognition and elimination of cancer cells targeted by PIT. Similarly, IFNs, which possess antiviral and immunomodulatory properties, have been considered in combination with PIT to augment the immune activation against cancer cells sensitized to light.^[^ ^74^ ^]^

However, the challenge lies in optimizing the delivery methods, dosages, and timing of cytokine administration alongside PIT to achieve the desired immune activation without causing excessive systemic toxicity.^[^ ^75^ ^]^ Cytokines play crucial roles in modulating immune responses. However, their systemic administration can lead to severe adverse effects, including cytokine release syndrome and autoimmune reactions. Therefore, it is essential to carefully balance the immune‐stimulating effects of cytokines with their potential for systemic toxicity when combined with PIT. Furthermore, while preclinical studies show promise in enhancing PIT efficacy by boosting the immune response against cancer cells, clinical translation and validation of these strategies in human trials are needed. Therefore, more research is required to fine‐tune the integration of cytokines with PIT, ascertain safety profiles, and determine the optimal combination protocols for improved therapeutic outcomes in cancer patients.

#### 2.2.5 TLR Agonists

TLR agonists are molecules that activate the innate immune system by binding to TLRs, initiating immune responses against pathogens or cancer cells.^[^ ^57^ ^]^ Integrating TLR agonists with PIT represents a promising strategy to enhance the immune response against tumors. Studies have investigated the use of TLR agonists alongside PIT to augment the immune‐mediated destruction of cancer cells. These agonists can be administered locally or systemically to the tumor site to engage TLRs and trigger immune activation in combination with phototherapies. For example, TLR‐7 and TLR‐8 agonists have been explored in preclinical models in combination with PIT.^[^ ^76^ ^]^ These agonists, when used in conjunction with the light‐activated compounds in PIT, effectively boost host immune responses against the sensitized cancer cells. TLR‐7 agonists, such as imiquimod, activate immune cells like DCs and induce the release of pro‐inflammatory cytokines.^[^ ^77^ ^]^ When combined with PIT, imiquimod heightens the immune response within the tumor microenvironment, potentially improving the overall efficacy of the treatment. Similarly, TLR‐9 agonists like CpG oligodeoxynucleotides have been investigated alongside PIT.^[^ ^78^ ^]^ These molecules trigger immune cell activation, promote the release of cytokines, and stimulate immune cell infiltration into tumors, potentially enhancing the immune‐mediated destruction of cancer cells.

The synergy between TLR agonists and PIT aims to create a more robust and sustained antitumor immune response. However, refining the dosages, delivery methods, and timing of TLR agonist administration in combination with PIT is crucial to optimize therapeutic outcomes while minimizing potential adverse effects. While preclinical studies show promise, clinical trials are needed to validate the safety, efficacy, and feasibility of combining TLR agonists with PIT in human cancer treatment. The understanding of complex interactions between TLR agonists and phototherapies remains an active area of investigation for developing more effective immunotherapeutic strategies against cancer.

#### 2.2.6 Adoptive T Cell Therapies

**Figure 5:**
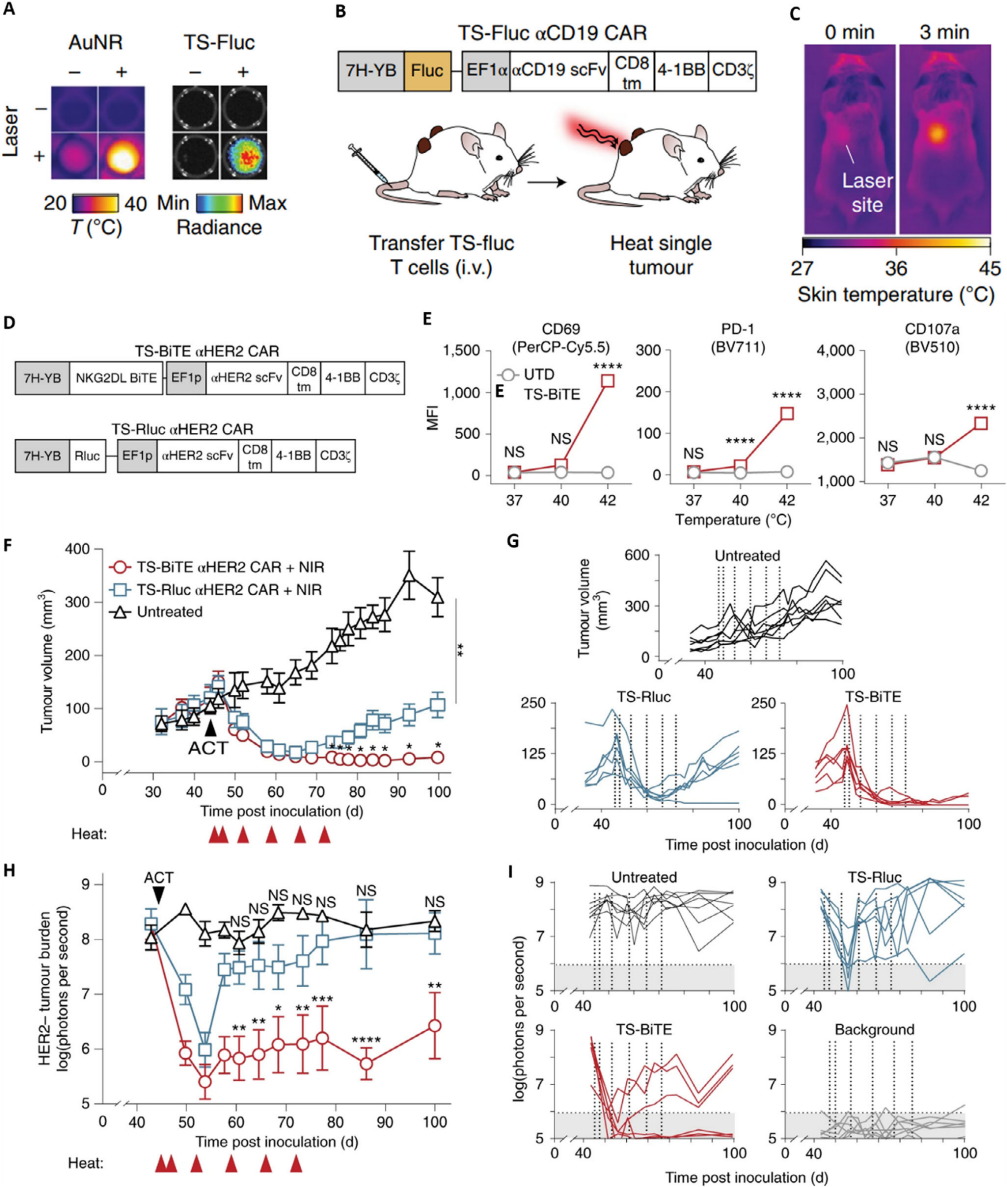
Photothermally controlled synthetic gene switches for controlling the activity of CAR‐T cells. A) Thermal and luminescence imaging of wells containing TS‐Fluc T cells post‐irradiation with a NIR laser. The thermal images on the left were taken using a FLIR thermal camera, while the luminescence images on the right were recorded 6 h after heating with an IVIS Spectrum CT system. B) A diagram showing the TS‐Fluc αCD19 CAR construct transduced into primary human T cells before their transfer into NSG mice with two flank tumors (either K562 or Raji), followed by photothermal heating of one of the tumors. C) Thermal images of a mouse captured during laser irradiation of the tumor site at 0 and 3 min. D) Schematic of TS‐BiTE and TS‐Rluc αHER2 CAR vectors. E) Flow cytometry quantification of activation markers CD69, PD‐1, and CD107a by TS‐BiTE αHER2 CAR T cells after 30 min heating and co‐culture with HER2–MDA‐MB‐468 target cells. F) Tumor growth curves of MDA‐MB‐468 tumors inoculated at a 3:1 HER2+ to HER2− ratio and treated with TS‐BiTE or TS‐Rluc αHER2 CAR T cells. Heat treatments were carried out on days 45, 47, 52, 59, 66, and 72. G) Spider plots of individual tumors, with vertical dotted lines indicating heat treatments. H) In vivo luminescence imaging time course. I) Individual spider plots acquired by an IVIS Spectrum CT system representative of the HER2−/Fluc+ cell population in the mixed MDA‐MB‐468 tumor model. Adapted with permission.^[^ ^80^ ^]^ Copyright 2021, Nature. Abbreviations: TS‐BiTE αHER2 CAR: CAR‐T cell construct designed for targeting HER2 positive cancers, TS‐BiTE αHER2 CAR + NIR: NIR light driven photothermal activation of TS‐BiTE αHER2 CAR, TS‐Rluc αHER2 CAR: tumor‐specific receptor‐luciferase with a CAR targeting HER2‐positive tumor cells, TS‐Rluc αHER2 CAR + NIR: NIR light driven photothermal activation of TS‐Rluc αHER2 CAR, UTD: untransduced groups, ACT: adoptive cell transfer, BiTEs: bispecific T cell engagers, IVIS: in vivo imaging system, CT: computed tomography.

While the direct integration of ACT with PIT is not extensively documented in clinical settings, the principles of combining these therapies hold promise in cancer research.^[^ ^58^ ^]^ In theory, modified T cells used in ACT could be engineered to express chimeric antigen receptors (CAR‐T cells) or T cell receptors targeting specific tumor antigens. These modified T cells, designed to recognize and attack cancer cells, could be administered to the patient. Subsequently, PIT could be employed to activate or enhance the function of these engineered T cells.^[^ ^28^ ^]^ The light‐based activation methods could be used to stimulate the modified T cells selectively within the tumor site, potentially increasing their cytotoxic effects against cancer cells. For example, in a preclinical study, researchers have explored combining CAR‐T cell therapy with a phototherapy.^[^ ^79^ ^]^ Typically, CAR‐T cell therapy yields poor responses in treating solid tumors, and systemic delivery of immunomodulatory biologics to augment CAR‐T cell activity often leads to off‐target toxicity, limiting their therapeutic potential. One of the studies demonstrates that the activity of CAR‐T cells within tumors can be controlled using photothermal methods through synthetic gene switches,^[^ ^80^ ^]^ **Figure** **5**. These gene switches are triggered by mild temperature elevations (40–42 °C) induced by photothermal agents or even a fever.^[^ ^81^ ^]^ However, the higher temperature (>50 °C) could cause necrosis and may not be helpful for controlled cell death.^[^ ^82^ ^]^ The in vitro experiments showed that mild heating of engineered T cells led to significantly higher expression of a reporter transgene without affecting their proliferation, migration, or cytotoxicity. In mouse models, CAR‐T cells heated photothermally via gold nanorods selectively produced the transgene within tumors. Additionally, systemic delivery of CAR‐T cells followed by intratumoral production of immunomodulatory agents under photothermal control further enhanced the CAR‐T cell activity against tumors and reduced the risk of antigen escape (i.e., losing or altering specific antigens). This localized photothermal control of engineered T cell activity holds promise for improving the safety and efficacy of CAR T cell therapy in treating solid malignancies. Furthermore, recent studies have demonstrated additional novel approaches, such as combining PDT with CAR‐T cells engineered to express photoactivatable cytokines like IL‐12, allowing light to modulate immune activity precisely within tumor sites.^[^ ^83^ ^]^ Another approach involves conjugating CAR‐T cells with upconversion nanoparticles, which convert near‐infrared light to visible light, thus activating light‐sensitive CAR‐T cells deep within tumors, offering a promising solution for solid tumor accessibility.^[^ ^84^ ^]^

These innovative strategies, though largely in preclinical phases, demonstrate the potential synergy between ACT and PIT in enhancing the precision and effectiveness of cancer treatment. The translation of these concepts into clinically viable treatments requires further research, including comprehensive preclinical investigations and eventually clinical trials, to validate the safety, efficacy, and feasibility of such combined approaches in cancer therapy.

#### 2.2.7 Small Molecules

There are different classes of small‐molecule drugs or compounds that have been investigated for their potential synergy with phototherapies.^[^ ^59^ ^]^ PI3K inhibitors, such as buparlisib,^[^ ^85^ ^]^ idelalisib,^[^ ^86^ ^]^ copanlisib,^[^ ^87^ ^]^ duvelisib,^[^ ^88^ ^]^ and alpelisib^[^ ^88^, ^89^ ^]^ have been explored in cancer treatment.^[^ ^90^ ^]^ These inhibitors target the PI3K pathway, and their combination with PIT might influence cellular responses to light‐induced cytotoxicity by several mechanisms. Firstly, inhibition of the PI3K pathway can sensitize tumor cells to oxidative stress‐induced cell death, enhancing the cytotoxic effects of ROS generated during PIT.^[^ ^91^ ^]^ Additionally, PI3K signaling is involved in cellular processes that contribute to tumor cell survival and resistance to therapy, such as apoptosis inhibition and DNA repair. By blocking these pro‐survival pathways, PI3K inhibitors increase the susceptibility of tumor cells to the cytotoxic effects of PIT, leading to enhanced tumor cell death.^[^ ^92^ ^]^ We are also working on combining the buparlisib with the lipid and polymer‐based NIR‐II photothermal agents and have found promising results for the PIT of solid tumors (unpublished). Kinase inhibitors, such as imatinib, erlotinib, sorafenib, sunitinib, etc., target various signaling pathways like MAPK, mTOR, or tyrosine kinases, and have also been studied alone or in combination with PIT.^[^ ^93^ ^]^ Their potential integration with PIT aims to modulate signaling pathways within cancer cells, potentially impacting their sensitivity to phototherapies by restricting tumor growth, angiogenesis, survival, and immunosuppression. Antiangiogenic agents, such as vascular endothelial growth factor inhibitors (e.g., bevacizumab, ramucirumab), target angiogenesis, and are known to impact the tumor microenvironment.^[^ ^94^ ^]^ Their combination with PIT can potentially affect tumor vasculature and oxygenation, influencing the efficacy of light‐induced cytotoxicity.^[^ ^95^ ^]^ Metabolic modulators, like PARP inhibitors or agents influencing mitochondrial function, target cellular metabolism or DNA repair pathways, and have been considered in combination with various cancer therapies.^[^ ^96^ ^]^ Their impact, in combination with PIT, remains an area of ongoing investigation.^[^ ^97^ ^]^

Besides these small molecules, several NIR light‐responsive biomaterials like gold^[^ ^60^ ^]^ nanoshells, nanorods,^[^ ^61^ ^]^ carbon nanotubes,^[^ ^62^ ^]^ and graphene^[^ ^63^ ^]^ also show the immunomodulatory properties after surface functionalization. The immunomodulators have been used alone or in combination with anticancer drugs or other immunomodulators to achieve the desired therapeutic efficacy in PIT.^[^ ^64^ ^]^

### 2.3 Photo‐Activable Agents

Photosensitizers or photothermal agents are molecules that absorb light energy and undergo photochemical reactions, playing a crucial role in PTT, PDT, and PIT. In PTT, photothermal agents convert light energy into heat through non‐radiative processes, leading to localized hyperthermia and subsequent cell death. In PDT, photosensitizers generate ROS upon light activation, inducing oxidative stress and cytotoxicity in target cells. PIT utilizes photo‐responsive/activable agents conjugated to targeting ligands, enabling selective binding to specific cell surface receptors, thereby facilitating targeted phototoxicity upon light exposure. These approaches exploit the precise spatiotemporal control of light activation to achieve therapeutic effects with minimal off‐target damage. The following are two major photoactivable agents that have been used in PIT. Indocyanine green (ICG): Though primarily known for medical imaging, ICG has been explored as a photo‐activable agent in PIT.^[^ ^98^ ^]^ It can be conjugated to antibodies or used in nanoparticle formulations to target specific cancer cells. Porphyrin‐based compounds: Compounds like photofrin or 5‐aminolevulinic acid are used in PDT and have been considered for their potential in PIT.^[^ ^99^ ^]^ These compounds accumulate in cancer cells and, upon light exposure, induce cell death.

### 2.4 Carrier

Researchers have designed carriers that can modulate the release of immunomodulators due to externally applied light.^[^ ^100^ ^]^ The NIR light‐responsive carriers allow the spatiotemporal control on the release of immunomodulant as well as help in minimizing the systemic and off‐target toxicity. Nanoparticles: Various nanoparticles, such as gold nanoparticles, silica nanoparticles, liposomes, etc., have been functionalized or loaded with photoactive compounds to serve as carriers for PIT. These nanoparticles can also be engineered to target specific cells or tissues. Additionally, polymer‐based carriers can encapsulate photosensitizing agents and enable controlled release of immunomodulators and/or anticancer drug(s) at the targeted site. For example, previously we have used a combinatorial approach for treating cancer in a murine model,^[^ ^4f,g^ ^]^ Figure 4B–E. Briefly, we used the α‐CD‐based gel system and bilirubin nanoparticles as the carrier for the immunomodulators (CpG and IND) as well as for the photothermal agents (ICG and chlorin) for realizing the combinatorial effect of PTT and immunotherapy. Antibody‐drug conjugates: Antibody‐drug conjugates (ADCs) offer a highly targeted approach in cancer therapy by merging Mab with photothermal dyes^[^ ^101^ ^]^ or photosensitizers.^[^ ^102^ ^]^ These conjugates are engineered to specifically bind to tumor‐associated antigens found on cancer cells. Once the ADC attaches to its target, it is taken up by the cancer cell, releasing the photothermal dye or photosensitizer inside. Photothermal dyes remain inactive until exposed to NIR light, which then generates localized heat to destroy the cancer cell through hyperthermia. Photosensitizers, on the other hand, produce ROS when activated by light, leading to cellular damage and cell death. This dual mechanism allows for precise targeting and destruction of cancer cells while sparing surrounding healthy tissues. The advantage of ADCs lies in their ability to control treatment effects spatially and temporally through light exposure. This precision reduces side effects and enhances treatment efficacy.

### 2.5 Mechanism of Cell Death and Specificity

**Figure 6:**
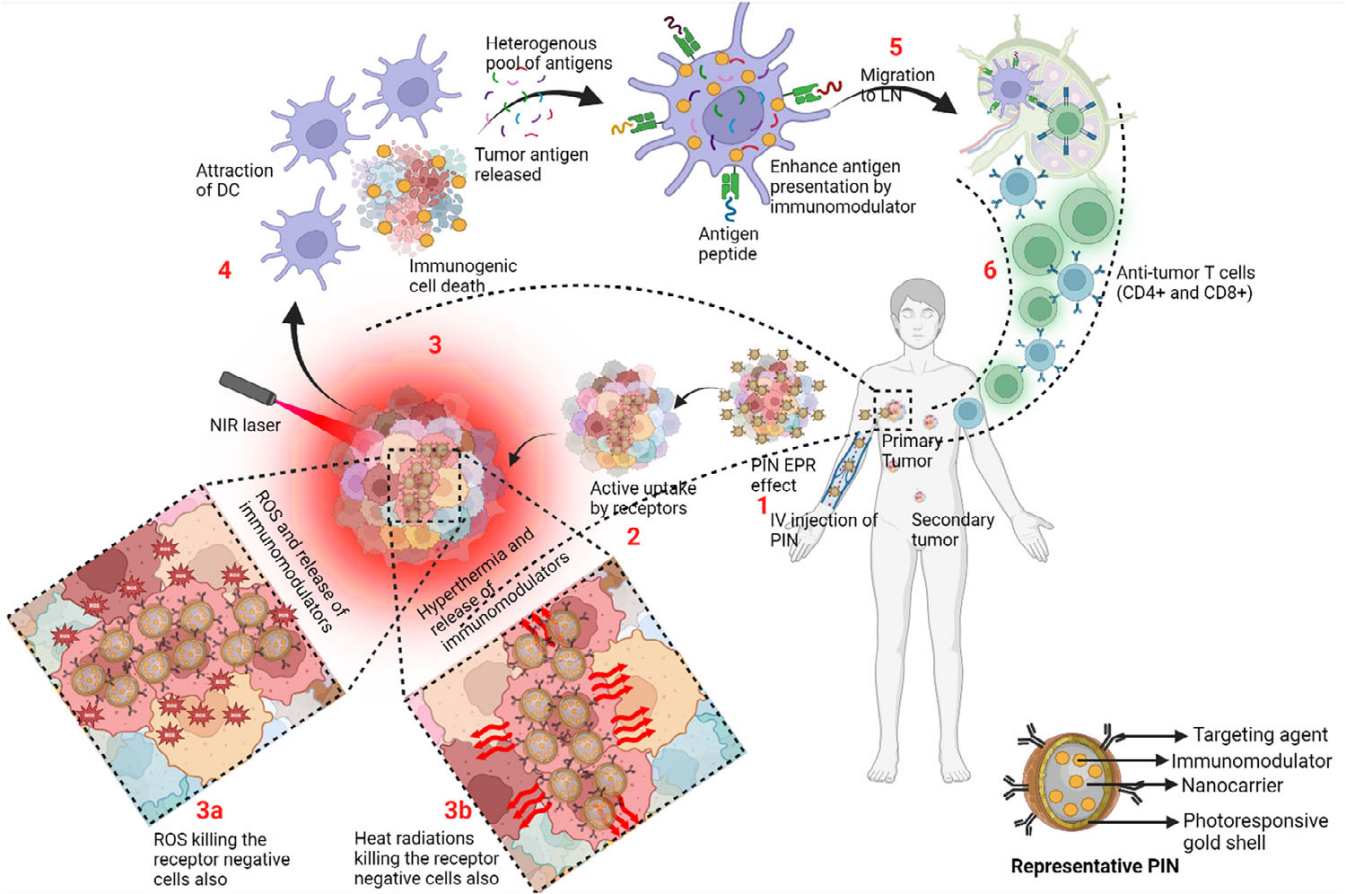
Mechanism of PINs driven photoimmunotherapy. 1) After intravenous administration of PINs, it reaches the primary tumor via the EPR effect. 2) It's actively taken up by the cancer cells with the help of targeting moiety. 3) Laser irradiation causes a) ROS generation or b) hyperthermia. 4) The ICD is due to ROS generation or hyperthermia, which causes the release of tumor antigens and immunomodulators loaded in PINs. 5) Immunomodulators help in enhancing the antigen presentation by the APC, which migrates to the lymph node. 6) Anti‐tumor immune response eliminates the secondary tumor and left out cancer cells in the body.

The general mechanism of PINs involves the selective delivery of immunomodulatory agents to specific cells or tissues, followed by the photoactivation of PINs upon exposure to light,^[^ ^103^ ^]^ **Figure** **6**. Upon activation, the immunomodulatory agents are released from PINs and then modulate the activity of immune cells, leading to an enhanced immune response against cancer cells. The irradiation of NIR light in the presence of PINs damages the integrity of the cell membrane of cancer cells and allows water to enter rapidly, leading to swelling, rupture, and cell death. The oxidative stress (PDT driven PIT), release of axial ligand, and hyperthermia (PTT driven PIT) have been found to be mainly associated with PINs‐driven cell death.

The oxidative stress has been related to necrosis during the PDT of cancer.^[^ ^104^ ^]^ However, how it is involved in the PDT driven PIT is debatable. Railkar et al. have shown the significant generation of ROS after the PDT for the treatment of bladder squamous cell carcinoma, wherein the ROS scavengers completely prevented cell death.^[^ ^105^ ^]^ However, the response was partly in the study of Jin et al. and Mitsunga in the treatment of MDA‐MB231 and 3T3/HER2 cells, respectively.^[^ ^106^ ^]^ In recent studies, mass spectrophotometer has been used to determine whether ROS‐driven oxidation of lipid molecules is the leading cause of membrane disruption‐driven cell death. It was found that the main component of the cell membrane, phosphatidylcholine, is minimally oxidized, thus suggesting a minimal role in the physical stress. Some studies even showed the enhancement of liposome disruption in deoxygenated conditions, suggesting another underlying mechanism along with or without oxidative stress.^[^ ^107^ ^]^ Additionally, ferroptosis and cuproptosis may play significant roles in PDT‐induced cell death. Ferroptosis, characterized by iron‐dependent lipid peroxidation and mitochondrial dysfunction, has been associated with elevated oxidative stress that leads to the destruction of cellular integrity.^[^ ^108^ ^]^ Dysregulation in iron metabolism initiates the Fenton reaction, resulting in free radical accumulation and cell death. Cuproptosis, on the other hand, is driven by the accumulation of copper within cells, leading to lipoylated protein aggregation, loss of iron‐sulfur cluster proteins, and oxidative stress, eventually triggering proteotoxic stress and cell death.^[^ ^109^ ^]^ However, more studies are required to further understand the oxidative stress on cell membranes due to ROS.

In the case of photothermal agents like IR700‐driven PIT, the mass spectrophotometry has shown that IR700 releases the axial ligand (C_14_H_34_NO_10_S_3_Si) after 690 nm laser‐driven irradiation.^[^ ^104^ ^]^ The PINs, such as Mab conjugated NIR dye (moAB‐IR700), become hydrophobic, which causes aggregation and thus leads to disruption of the transmembrane osmotic gradient. It causes the swelling and bursting of cells. This phenomenon has also been demonstrated by the 3D low coherence quantitative phase microscopy and dual view inverted selective plane illumination microscopy.^[^ ^6^ ^]^ There is also a theoretical explanation for the release of axial ligand from IR700. For example, Kobayashi et al. have proposed that after the release of radical anion from IR700, it is attacked by the water molecule, which helps to clear the central Si─O bond of IR700 via protonation reaction.^[^ ^110^ ^]^ The release of axial ligand leads to damage to cancer cells. It was supported by Anderson et al., wherein the death of nearby non‐irradiated cells was shown after the 690 nm laser activation of silicon phthalocyanine in hypoxic conditions.^[^ ^111^ ^]^ It indicates that axial ligand release could be the primary mechanism of NIR dye‐based PTT driven PIT for killing cancer cells in hypoxic conditions, wherein ROS mediated killing effects are compromised.

The specificity and selectivity of PINs are achieved by targeting the overexpressed receptors on the surface of cancer cells and by the exclusive responsiveness of PINs to NIR lasers.^[^ ^104^ ^]^ This leads to little or no damage to the surrounding normal cells.^[^ ^106^, ^112^ ^]^ Additionally, the targeted cell death due to PINs is immunogenic because cellular components are released, including cancer cell‐specific antigens.^[^ ^6^ ^]^ The PINs‐driven PIT can also upregulate the different heat shock proteins like HSP70 and HSP90, which can bind with the tumor antigens and interact with TLRs to activate APCs. Also, the release of calreticulin, HMGB1, and ATP helps in promoting the maturation of DCs in the tumor microenvironment.^[^ ^113^ ^]^ The mature DCs help in educating and proliferating the cytotoxic T cells toward the cancer cell‐specific killing, which is in contrast to many cancer therapies that are apoptotic but not very helpful in promoting ICD. For instance, chemotherapy and radiotherapy kill the cancer cells but also severely affect immune cells.^[^ ^114^ ^]^ While in PINs‐driven PIT, multiple neoantigens are released, which helps in priming and proliferation of CD8+ T cells toward the specific killing of cancer cells throughout the body, also known as the abscopal effect.^[^ ^115^ ^]^ Further, NIR light does not use ionizing radiation and is applied in nanothermal dosage, so there is no damage to cellular DNA. In most preclinical studies, the animals treated with PINs show significant tumor shrinkage with very few adverse effects.^[^ ^116^ ^]^ Also, unlike other conventional therapies for cancer, it can be repeatedly administered if relapse or residual cancer cells remain. The repeated dosing of PINs has been shown to improve the efficacy of PIT, leading to a complete response in solid tumors.^[^ ^25^ ^]^

In summary, PINs are designed for enhanced tumor targeting using optimized size, shape, and surface modifications. They integrate immunomodulators and photoactivable agents for controlled drug release and immune activation. Their mechanism involves photoactivation‐induced ROS, hyperthermia, and ICD, promoting a systemic anti‐tumor response. Overcoming toxicity, stability, and scalability challenges is key to clinical translation.

## 3 Clinical Side of Phototherapies and Immunotherapy

**Figure 7:**
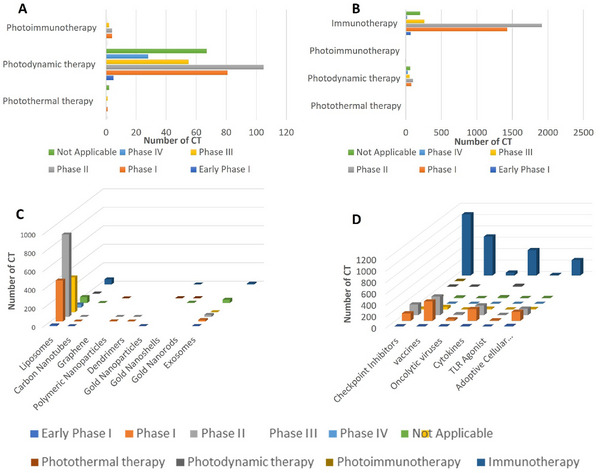
Number of clinical trials and their phases in different cancer therapies. A) Number of different clinical trials (CT) under nanomedicines driven phototherapies, i.e., photothermal, photodynamic, and photoimmunotherapy. B) Comparison of clinical trials in phototherapies to immunotherapy. C) Number of clinical trials under the head of different clinically viable nanomaterials. D) Number of clinical trials under the head of different clinically viable immunomodulators. *Source*: Generated with the information from clinicaltrials.gov.

Since the start of cancer nanomedicines, continuous improvements such as prolonged survival, reduced toxicity, and an increase in response rate have been observed over the past decades. However, there is still significant prolongation in the clinical translation and many controversies related to nanomedicines‐driven PDT, PTT, and PIT when compared to immunotherapy for cancer, as shown in **Figure** **7**A,B. Herein, we review and compare the clinically relevant photo‐responsive/activable nanocarriers, immunomodulators, modes of administration, and targeting agents used in clinical trials of either cancer therapy. To curate the clinical trial data, we searched the portal clinicaltrials.org using various keywords under the cancer category.

### 3.1 Clinically Relevant Photo‐Responsive/Activable Nanocarriers

There are several clinically relevant photo‐responsive/activable nanocarriers that have been investigated for PDT, PTT, and PIT applications, Figure 7C. These nanocarriers can be activated by light to release their payload, which includes immunomodulatory agents.

Liposomes, lipid‐based vesicles capable of encapsulating hydrophilic and hydrophobic molecules, can be transformed into photo‐responsive/activable carriers by incorporating NIR light‐responsive dyes or photothermal agents or by coating their surface with gold.^[^ ^117^ ^]^ These modified liposomes are designed to release their cargo, such as immunomodulants, upon exposure to specific wavelengths of light.^[^ ^118^ ^]^ This carrier has been harnessed in PDT, PTT, and PIT. Notably, the clinical landscape reflects the growing interest, with 1822 liposome‐based clinical trials registered on clinicaltrials.gov, Figure 7C. Of these, a significant number are dedicated to immunotherapy and PDT, with 443 studies in Phase I and 35 in Phase IV, respectively.

For PDT, liposomes allow for precise delivery of photosensitizers, enhancing therapeutic outcomes with light activation, but their stability issues can lead to premature release before reaching the tumor site. In PTT, their ability to incorporate photothermal agents like gold nanoparticles makes them effective for localized heat generation, but their complex production and cost remain barriers. For PIT, liposomes can be modified to release immunomodulators in response to specific wavelengths, aiding immune activation, although potential immunogenicity and drug leakage can limit their efficiency.

An example of liposomes used in PDT is the Visudyne formulation, which encapsulates the photosensitizer verteporfin. This system is activated by light to treat age‐related macular degeneration but has also been explored for cancer PDT due to its controlled release and selective targeting of cancerous tissues. For PTT, liposomes have been coated with gold nanoparticles, such as in studies using DOX‐loaded gold‐coated liposomes, where gold's photothermal properties enable enhanced drug delivery and localized heating for tumor ablation. In PIT, liposomes encapsulating immunomodulatory agents like CpG oligodeoxynucleotides are light‐triggered to boost the immune response against tumors, improving therapeutic outcomes in combination with immune checkpoint inhibitors.

Polymeric nanoparticles, synthesized using FDA‐approved polymers like polylactic acid and poly lactic‐co‐glycolic acid, can also be engineered to respond to light stimuli. The incorporation of photothermal and/or photodynamic agents allows these polymeric nanoparticles to render PTT and/or PDT‐driven PIT along with the controlled release of immunomodulator(s).^[^ ^4^, ^119^ ^]^ Currently, only four clinical trials feature polymeric nanoparticles for cancer treatment, three of which remain in Phase I, and one has advanced to Phase II, Figure 7C. There are no polymeric nanoparticle‐based clinical trials have been registered for any of the discussed therapies in this review.

For PDT, an example is the use of methoxy poly(ethylene glycol)‐b‐poly(d,l‐lactide) (PEG‐PLA) nanoparticles encapsulating the photosensitizer Zn(II)‐phthalocyanine, which offers enhanced stability and targeted release upon light exposure. In PTT, polymeric nanoparticles like PLGA nanoparticles loaded with ICG are known for their efficient light‐to‐heat conversion, which enhances tumor destruction while minimizing damage to surrounding tissues. For PIT, PLGA nanoparticles loaded with immunoadjuvants can be light‐triggered to release immunomodulatory drugs, stimulating immune system activation and improving therapeutic effects in cancer immunotherapy.

In PDT, polymeric nanoparticles provide precise control over the release of photosensitizers, reducing systemic side effects, but their complex and costly manufacturing limits scalability. In PTT, they can be loaded with photothermal agents for effective tumor ablation through heat, but their use in clinical settings remains limited due to a lack of robust clinical data. For PIT, polymeric nanoparticles can offer controlled immunomodulator release upon light exposure, enhancing immune responses, though their slow progression through clinical trials delays their adoption.

Gold nanoparticles, in the form of different structures like nanorods and nanostars, possess exceptional optical properties, facilitating the conversion of light energy into heat through photothermal conversion.^[^ ^120^ ^]^ This property enables the triggering of immunomodulant release from nanocarriers and/or ICD upon laser light exposure.^[^ ^121^ ^]^ However, only 4 clinical trials based on gold nanoshells are registered on clinicaltrials.gov, and also their pilot nature prevents them from being assigned a specific phase status. Currently, there are 2 gold nanoparticle‐based clinical trials for the PTT and 1 for the immunotherapy, Figure 7C.

For PDT, gold nanoparticles can enhance the delivery and activation of photosensitizers due to their optical tunability, increasing the effectiveness of light‐induced tumor destruction, but concerns about their long‐term safety and potential toxicity from accumulation remain. In PTT, gold nanoparticles are highly effective at converting light to heat for precise thermal ablation of tumors, though their high production costs and potential environmental impact limit their widespread use. For PIT, gold nanoparticles can efficiently release immunomodulators upon laser exposure, aiding immune stimulation, but their limited clinical trials and concerns about potential off‐target effects have restricted their clinical application.

In PDT, gold nanoclusters conjugated with porphyrin have been explored as potent photosensitizers, with their enhanced light absorption boosting ROS generation to kill cancer cells. For PTT, a widely studied example is AuroShell nanoparticles (gold nanoshells), which convert light energy into heat, effectively ablate tumors while minimizing damage to healthy tissues. In PIT, gold nanorods conjugated with anti‐PD‐L1 antibodies are used to release immunomodulators upon NIR light exposure, facilitating localized immune activation and improving the therapeutic response to checkpoint inhibitors.

Carbon nanotubes are endowed with excellent light‐absorbing capabilities, generating heat when exposed to light.^[^ ^122^ ^]^ As carriers for immunomodulants, they can release their cargo upon light activation. The clinical trials involving graphene‐based carriers are limited, with one in Phase I and one in Phase II, while another does not possess a designated phase, Figure 7C. In terms of therapy, only 1 clinical trial of graphene for the PTT, while no one is registered for the PDT, PIT, and IT.

It is imperative to recognize that while these nanocarriers exhibit promise, their transition to clinical use necessitates rigorous safety and efficacy testing. The regulatory approval processes are paramount in determining the clinical viability of these nanomaterials.

In PDT, carbon nanotubes offer a large surface area for loading photosensitizers, improving the efficiency of light‐triggered drug release, but their potential toxicity and accumulation in tissues pose significant safety concerns. In PTT, carbon nanotubes have excellent light absorption and heat generation properties, making them powerful tools for thermal ablation, though issues with biodegradability and regulatory approval hinder their clinical adoption. For PIT, carbon nanotubes can be used to deliver immunomodulators with light‐triggered release, but the lack of extensive clinical testing and concerns about long‐term toxicity limit their use in immune‐based cancer therapies.

For PDT, single‐walled carbon nanotubes (SWCNTs) functionalized with photosensitizers such as porphyrins have shown great promise due to their high surface area and ability to target tumors efficiently. In PTT, multi‐walled carbon nanotubes (MWCNTs), when combined with NIR light, generate significant heat to destroy tumor cells, with several preclinical studies demonstrating their effectiveness. For PIT, carbon nanotubes functionalized with immunomodulatory agents such as TLR agonists can release their cargo upon light activation, enhancing the immune system's response to cancer cells.

Exosomes have emerged as a promising nanocarrier system in cancer therapy, particularly in immunotherapy and PIT due to their natural biocompatibility, immune‐modulatory properties, and ability to efficiently deliver therapeutic payloads, including immunomodulators and photosensitizers.^[^ ^123^ ^]^ Unlike synthetic nanoparticles, exosomes are naturally derived extracellular vesicles that facilitate intercellular communication and can be engineered to enhance immune activation or tumor targeting. Clinical trials investigating exosome‐based therapies are expanding, with 2 studies in early‐phase trials, 17 in Phase I, 22 in Phase II, and 2 in Phase III, reflecting growing interest in their translational potential. Additionally, 36 studies fall under non‐applicable phases, likely including exploratory or observational studies. While PIT‐specific clinical trials are not yet registered, 10 clinical trials in immunotherapy indicate the growing role of exosomes in modulating immune responses, potentially synergizing with phototherapies. Given their ability to encapsulate and transport bioactive molecules while evading immune clearance, exosome‐based PINs hold significant promise for improving targeted delivery and enhancing the efficacy of PIT and immunotherapy in clinical oncology.

### 3.2 Clinically Relevant Immunomodulant

The major classes of immunomodulators that are commonly being studied in clinical trials for various cancer types include checkpoint inhibitors, vaccines, adoptive cellular immunotherapy, OV, TLR agonists, and cytokines Figure 7D.

Checkpoint inhibitors function by blocking the inhibitory receptors like CTLA‐4 and PD‐1, which typically restrict the activity of T cells.^[^ ^124^ ^]^ This action eliminates suppression, allowing existing tumor‐specific T cells to proliferate, act as effector cells, and fight against cancer cells. Notably, checkpoint inhibitors such as nivolumab and pembrolizumab have gained approval for numerous cancer types, whether employed individually or in combination.^[^ ^125^ ^]^ The current landscape showcases 362 clinical trials exploring checkpoint inhibitors in cancer treatment, with a substantial portion (187) in Phase II. There are only 3 clinical trials of checkpoint inhibitors for PDT and 2 for PIT; however, 1071 trials are registered for immunotherapy, Figure 7D.

Cancer vaccines aim to trigger adaptive anti‐tumor immunity by loading DCs with tumor antigens.^[^ ^54^ ^]^ This can occur through ex vivo exposure to peptides/proteins or in vivo generation from mRNA/DNA vaccines, initiating antigen presentation, and activating naive T cells that subsequently migrate to tumor sites. An extensive array of 728 clinical trials exists for cancer vaccines, comprising 341 in Phase I and 327 in Phase II. Currently, there is only 1 clinical trial of cancer vaccine with PDT, while 682 with immunotherapy, Figure 7D. However, no clinical trials have been reported with PTT and PIT.

Adoptive cell therapies harness genetically engineered T cells provided with CAR that target tumor cells directly or T cell receptors designed to recognize tumor antigen‐MHC complexes. This prompts T‐cell signaling, cytokine release, and direct killing upon antigen recognition. CAR‐T cell therapies like tisagenlecleucel and axicabtagene ciloleucel have secured approval for blood cancers.^[^ ^126^ ^]^ The clinical landscape includes 293 trials, primarily distributed in phase I (159) and phase II (114), Figure 7D. There are no clinical trials of adoptive T cell therapy with PTT, PDT, and PIT. In comparison, 268 trials are registered with immunotherapy.

Oncolytic viruses selectively replicate within and rupture tumor cells, releasing viral progeny and tumor antigens that trigger direct cytotoxicity and the recruitment of innate and adaptive immune effector cells through ICD.^[^ ^127^ ^]^ Talimogene laherparepvec has gained approval for melanoma treatment.^[^ ^128^ ^]^ The clinical domain encompasses 48 trials, with 26 exclusively in Phase I, Figure 7D. There are no clinical trials of OV with PTT, PDT, and PIT, while 48 trials are registered with immunotherapy.

Other clinically relevant immunomodulators include cytokines, chemokines, and TLR agonists. These agents foster DCs and myeloid cell maturation, enhance T cell migration and function, and activate intracellular pathways that incite immune responses. The domain of cytokines holds 413 clinical trials, with 207 in Phase I and 170 in Phase II, Figure 7D. There are 16 clinical trials related to cytokines for the PDT, 444 for immunotherapy, and no one registered for the PTT and PIT.

### 3.3 Routes of Administration Used in Clinical Trials

**Figure 8:**
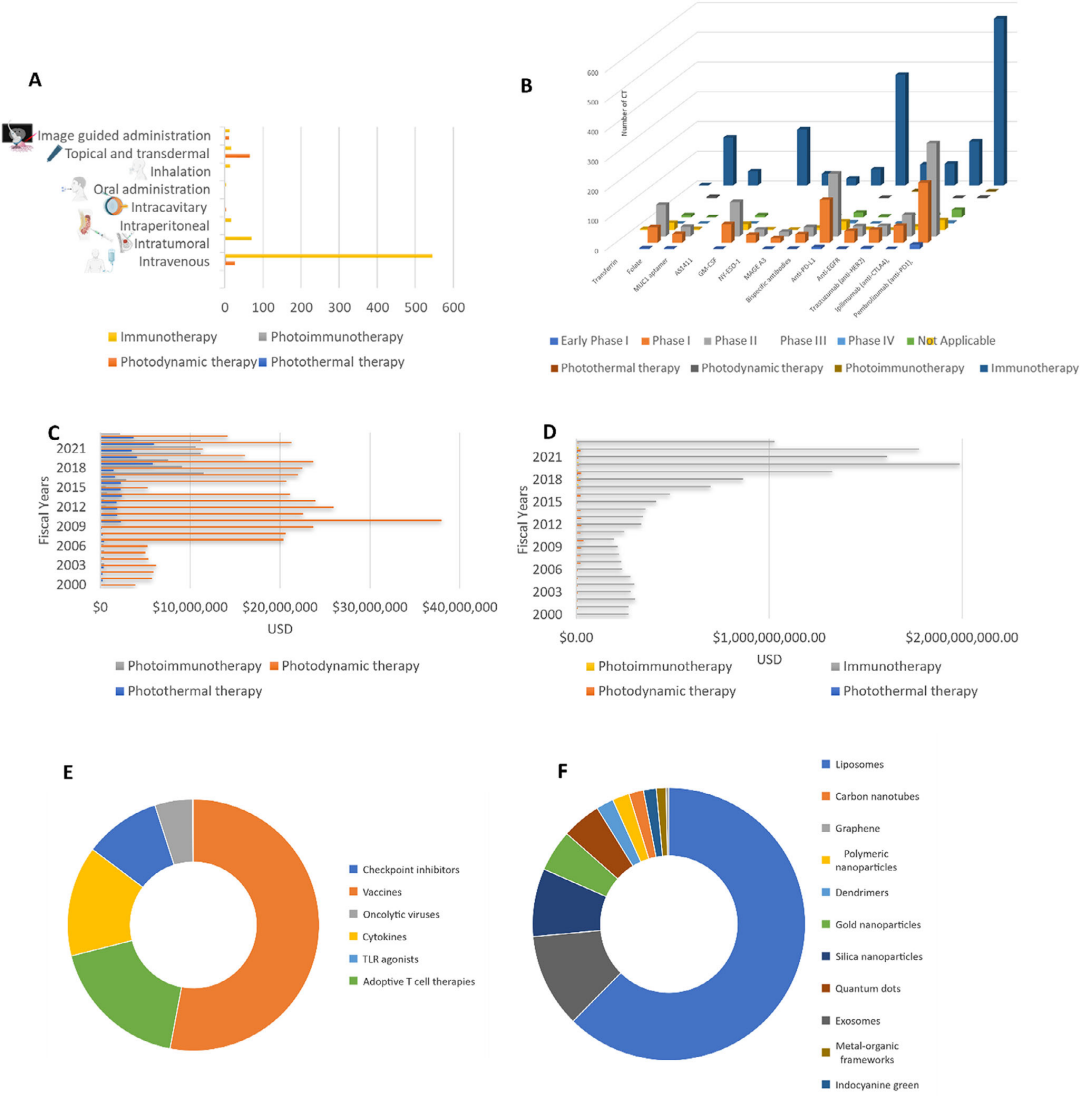
Clinical trial distribution by administration routes, targeting agents, and funding dynamics. A) The number of clinical trials following different routes of administration. IV administration is the most common route for PTT, immunotherapy, and photoimmunotherapy, while topical administration predominates in PDT. B) The use of clinically used targeting agents. Anti‐PD1 and anti‐PD‐L1 are the most frequently used targeting agents, with most trials in phase II. GM‐CSF and folate follow as the second most utilized immunomodulators, with over 100 clinical trials in phase II. C) The financial landscape of photoimmunotherapy, including the distribution of funds to different light‐driven cancer therapies. D) Comparison of funding for phototherapies versus immunotherapy. E) Funding allocation under major heads of clinically viable immunomodulators. F) Funding allocation under major heads of clinically viable nanomaterials for photoimmunotherapy. *Source*: Data generated using information from clinicaltrials.gov and NIH Reporter. Abbreviations: MUC1: mucin‐type 1 glycoprotein, AS1411: nucleic acid against nucleolin, GM‐CSF: cytokine adjuvant, NY‐ESO‐1: cancer‐testis antigens, MAGE A3: Melanoma‐associated antigen A3.

The clinical trials involving immunotherapy, PDT, PTT, and PIT for cancer treatment encompass diverse administration routes to deliver nanocarriers containing immunomodulators and photo‐responsive/activable agents to tumor sites, **Figure** **8**A. The selection of the administration route relies on factors such as type of the therapy and nature of nanomedicines, tumor location, therapeutic objectives, and patient comfort. Standard administration methods include intravenous (IV), intratumoral (IT), and topical applications.

#### 3.3.1 Intravenous Administration

PINs can be injected into the bloodstream, utilizing the EPR effect to achieve widespread distribution and accumulation in tumor tissues. Among 575 IV clinical trials, the majority (544) are practiced in immunotherapy, while 26 are focused on PDT, 3 on PIT, and 2 on PTT, Figure 8A. IT injection: Directly injecting PINs into tumors aims for high local immunomodulant concentrations while minimizing exposure to healthy tissue. This strategy is suitable for accessible localized tumors, with 75 ongoing intratumoral trials. Among these, 71 practiced immunotherapy, 2 targeted PDT, and 1 focused on PTT. Topical application: It targets skin cancers or lesions through topical application or transdermal delivery. Out of the 83 ongoing clinical trials, 17 centered on immunotherapy, while 66 explored PDT. Image‐guided administration: It employs imaging modalities like ultrasound, MRI, or CT for precise delivery of therapeutic modalities for immunotherapy, PTT, PDT, and PIT. Among the 24 ongoing clinical trials, 12 are practiced in immunotherapy, 11 in PDT, and 1 in PTT.

Additionally, various other routes, such as intraperitoneal administration, intracavitary administration, oral administration, and inhalation, are also explored in these therapies. On overviewing the scenario of the route of administration, it seems the selection of the administration route significantly influences the distribution and accumulation of therapeutic agents, which in turn affects the efficacy of these therapies. Each approach has its merits and limitations, necessitating careful consideration during the design and execution of PINs clinical trials for effective cancer treatment.

### 3.4 Targeting Strategies of PINs

PINs offer a promising avenue for targeted and precise control of immune responses. The following are some common targeting strategies.

#### 3.4.1 Active Targeting

PINs can be engineered to exhibit site‐specific targeted accumulation within tumors. Surface modifications involving ligands, antibodies, peptides, or aptamers that possess affinity for receptors or markers expressed on target cells can effectively amplify PINs accumulation and interactions with immune cells in the localized area. Several targeting agents are employed in PTT, PDT, PIT, and immunotherapy clinical trials, Figure 8B. For instance, antibodies like anti‐PD1, anti‐PD‐L1, anti‐CTLA4, anti‐HER2, and anti‐epidermal growth factor receptors (EGFR) are widely employed. Currently, antibody‐based immunotherapy clinical trials account for 1312 studies, with significant numbers for anti‐PD1 and anti‐PD‐L1, Figure 8B. Among these, anti‐PD1 is represented in 313 clinical trials in Phase II, while anti‐PD‐L1 counts up to 210 clinical trials. Also, there are only 2 anti‐PD1, 1 anti‐PD‐L1, and 1 anti‐CTLA4 study on PDT, 1 anti‐PD1 study, and 2 anti‐EGFR studies on PIT, while 561 anti‐PD1, 148 anti‐CTLA4, 73 anti‐HER2, 70 anti‐EGFR, and 372 anti‐PD‐L1 studies on immunotherapy. Most of the checkpoint inhibitor studies are on immunotherapy, some on PDT and PIT, while no clinical trials on PTT.

Furthermore, peptides and proteins such as MAGE A3 (antigen), NY‐ESO‐1 (antigen), and GM‐CSF (cytokine) also find application in immunotherapy‐focused clinical trials, Figure 8B. Among these, GM‐CSF leads with 204 clinical trials, 115 of which are in Phase II. Aptamers, including widely utilized ones like MUC1, also serve as targeting agents. MUC1, a prevalent aptamer, features in 66 ongoing clinical trials, with 32 in Phase II. Small molecules like folate and transferrin have also found roles in immunotherapy‐based clinical trials. Folate‐based clinical trials tally at 191, with 106 in Phase II, while transferrin‐based trials count at 3. Moreover, bispecific antibodies are increasingly integrated into immunotherapy‐based clinical trials, currently totaling 68 studies. Among these, 31 trials are practiced in Phase II, while 28 in Phase I. Of these immunomodulators except antibodies, only folate is tested in other treatment modalities other than immunotherapy, like 4 clinical trials in PDT.

#### 3.4.2 Passive Targeting

PINs can exploit the EPR effect, which allows them to accumulate in tumor tissues due to their leaky vasculature. The PINs‐driven PIT also shows effect on the vascularity of the tumor. Most of the tumors show the enhanced permeability and retention (EPR) effect due to improper blood vessel formation in the tumor microenvironment. However, after the PINs‐driven PTT, a rapid increase in vascularity has been observed in the tumor microenvironment.^[^ ^129^ ^]^ The quick killing of the perivascular cells in the tumor microenvironment because of innate PTT and PDT helps in enlarging the blood vessels due to an increase in space, thus favoring the reduction of vascular resistance. It helps enhance the EPR effect in comparison to the normal EPR effect. It has been noted that the increase in EPR could be up to 24 folds, which in general is less than 2%. It's also being named as super enhanced permeability and retention effect (SUPR).^[^ ^26^ ^]^ The improvement in efficacy has been reflected in the CD44 targeted anti‐PD‐1 driven PINs compared to the monotherapy.^[^ ^130^ ^]^ The SUPR effect helps in the uniform distribution of antibodies throughout the tumor.^[^ ^131^ ^]^ We haven't found any clinical trials with the EPR effect or that are solely based on passive targeting. Also, recent insights into nanoparticle tumor entry have revealed that both passive and active transport mechanisms play critical roles in nanoparticle delivery. While the EPR effect emphasizes passive targeting via inter‐endothelial gaps, recent studies suggest that active transport mechanisms, including transcytosis via nanoparticle‐transport endothelial cells, immune cell migration, and vesiculo‐vacuolar organelles, also significantly contribute to nanoparticle entry into tumors.^[^ ^132^ ^]^ These findings suggest that nanoparticle delivery to tumors may be more active than previously thought, with the active transport and retention (ATR) principle proposing that nanoparticles enter tumors primarily through active mechanisms and are retained by interactions with tumor‐associated macrophages and other tumor cells. Both EPR and ATR mechanisms can coexist, with their relative contributions depending on the tumor type, which adds complexity to the effectiveness of nanoparticle‐based therapies.

#### 3.4.3 Immunomodulation Timing

The ability of PINs to release immunomodulators in response to NIR light can also be used to target the release at precise locations and times. For instance, if the PINs are localized to a tumor site, exposing the tumor to NIR light could induce a controlled release of the immunomodulators, enhancing the local immune response against the tumor. PINs can also be tuned to release immunomodulators at specific times during the immune response.^[^ ^133^ ^]^ For example, releasing immunomodulators at the right moment can either suppress or enhance immune activity as needed, such as inhibiting excessive inflammation or promoting immune activation against tumors. Toward this, Dou et al. have developed a light‐sensitive optogenetic tool, LiSmore, to control cGAS‐STING signaling and selectively enhance immune responses.^[^ ^134^ ^]^ By activating LiSmore with light, they achieved precise dendritic cell maturation and improved T cell responses against tumors, creating a lasting antitumor effect. In a lung cancer model resistant to typical immune checkpoint therapies, LiSmore enhanced treatment outcomes and even suppressed tumor growth at a distant site in a mouse model of melanoma, highlighting the potential of targeted optogenetic activation in immune modulation.

#### 3.4.4 Personalized Medicine

Tailoring the design of PINs and choice of immunomodulator based on individual patient characteristics, such as tumor markers or genetic makeup, can also enhance the precision and effectiveness of the treatment. This personalized approach allows for the development of customized therapies that can adapt to the heterogeneity of tumors, addressing variations in tumor biology and patient responses.

#### 3.4.5 PINs in Clinical Trials

In this section, we have searched for laser‐assisted immunotherapy / PIT using clinicaltrial.gov and other available internet resources. In total, we found 14 clinical trials (**Table** **2**), out of which early phase I (1), phase I (6), phase II (5), and phase III (2). In phase I studies, out of 6 studies, 2 have been completed, and 1 is recruiting. Ongoing clinical trials on PIT provide critical insights into its potential and the challenges associated with its clinical translation. Several trials have explored PIT in combination with checkpoint inhibitors, and other immunotherapeutic agents to enhance treatment efficacy. Notably, ASP‐1929, a near‐infrared light‐activated antibody‐dye conjugate, has been the focus of multiple trials for head and neck squamous cell carcinoma. Trials such as NCT05265013 and NCT05182866 investigated its use alone or in combination with Pembrolizumab (PD‐1 inhibitor), assessing tumor response and survival outcomes. However, NCT05265013 was terminated, highlighting challenges that may be in safety, efficacy, and/or patient recruitment, while NCT05182866 remains active, exploring PIT's potential in pre‐surgical settings. Similarly, RM‐1995, another PIT agent targeting cutaneous squamous cell carcinoma and head and neck squamous cell carcinoma, was tested in NCT05220748, but the trial was withdrawn, emphasizing difficulties may be in dose optimization and/or regulatory approval.

**Table 2:** Overview of clinical trials under photoimmunotherapy.

#	Phase	NCT number	Composition (tradename)	Irradiation details	Indication (s)	Developer	Outcome	Status
1	III	NCT03202446	1% glycated chitosan and cyclophosphamide	No details provided	Breast cancer	Eske Corporation	No result posted	Terminated
2	I	NCT00453050	Imiquimod and indocyanine green	No details provided	Melanoma	University of Oklahoma	No result posted	Completed
3	Early phase I	NCT04570683	Nivolumab (opdivo)	Ablative fractionated laser	Basal cell carcinoma	Bispebjerg Hospital	No result posted	Recruiting
4	I and II	NCT03993678	IP‐001	TRANBERG laser irradiation for 30 min	Advanced solid tumors	Swiss Group for Clinical Cancer Research and Immunophotonics, Inc.	No result posted	Recruiting
5	NA	NCT02372708	Dinitrophenyl	Laser irradiation for 10 min, at a power density of 1 W cm^−2^	Melanoma	Chinese People's Liberation Army General Hospital	No result posted	Completed
6	II	NCT04400539	Nivolumab	Laser irradiation at a wavelength of 400–500 nm applied for 15 min at a dose of 25 J cm^−2^	Malignant Pleural mesothelioma	University Hospital, Lille	No result posted	Recruiting
7	I	NCT03341806	Avelumab	Laser interstitial thermal therapy	Glioblastoma	Icahn School of Medicine at Mount Sinai	No result posted	Completed
8	I	NCT00758797	Topical application of an immuno‐modulating cream	Diomed laser	Metastatic melanoma	Northwestern University		Terminated
9	II	NCT05265013	ASP‐1929, along with Pembrolizumab	PIT690 laser system		Rakuten Medical, Inc.	No result posted	Terminated
10	II	NCT05182866	ASP‐1929	PIT690 laser system	Head and neck cancer	Rakuten Medical, Inc.	No result posted	Active, not recruiting
11	III	NCT03769506	Compared ASP‐1929 with docetaxel, cetuximab, methotrexate, and paclitaxel	No details provided	Head and neck cancer	Rakuten medical, Inc.	No result posted	Recruiting
12	I and II	NCT04305795	Pembrolizumab and Cemiplimab. Additionally, ASP‐1929 is given intravenously on Day 8 of each cycle.	No details provided	Squamous cell carcinoma of the following types: recurrent head and neck, metastatic head‐and‐neck, locally advanced cutaneous, and metastatic cutaneous	Rakuten Medical, Inc.	No result posted	Active, not recruiting
13	I/IIa	NCT02422979	RM‐1929	690 nm	Recurrent head and neck cancer	Rakuten Medical, Inc.	Publication	Completed
14	I	NCT05220748	RM‐1995 and Pembrolizumab	PIT690 laser system	Squamous cell carcinoma of the following types: cutaneous, head and neck	Rakuten Medical, Inc.	No result posted	Withdrawn

In another approach, NCT04570683 investigated the combination of ablative laser therapy with Nivolumab, aiming to enhance immune activation and tumor antigen presentation. However, its status remains uncertain reflecting the broader issue of translating promising preclinical findings into clinical benefits. Meanwhile, in NCT04400539, researchers are evaluating the combination of PDT and immunotherapy for pleural mesothelioma, aiming to improve tumor regression through light‐activated immune modulation, though long study durations (2022 to 2026) pose further delays in clinical validation.

PIT holds significant potential for cancer treatment, but their clinical translation remains challenging due to regulatory hurdles, patient recruitment difficulties, and safety concerns such as phototoxicity and immune overactivation. Various nanocarriers and immunomodulators are currently under investigation in clinical trials, each with distinct advantages and limitations. Targeting strategies, including active ligand binding, EPR exploitation, and timed immunomodulation, aim to enhance therapeutic efficacy and improve tumor‐specific activation. However, the variability of EPR‐mediated nanoparticle accumulation, the complexity of combination regimens, and the lack of standardized treatment protocols further hinder clinical adoption. While PIN‐based therapies remain in the early stages of clinical trials, ongoing research, and technological advancements continue to support their potential as next‐generation cancer treatments. Despite the promise of PIT in checkpoint inhibitor synergy and tumor‐selective activation, its widespread clinical integration will require refined dosing strategies, improved light‐delivery methods, and stronger regulatory frameworks to ensure long‐term viability as a mainstream cancer therapy.

## 4 PINs Development—Market and Government Financing

The global market for PIT is anticipated to reach $309 billion by 2030, according to reports from various sources and ongoing clinical trials, Figure 8. However, before discussing financing landscape in detail, its comparison with other phototherapies and immunotherapy in terms of government financing is important to understand its potential impact on the healthcare industry. Figures 8C and D present NIH funding allocation across immunotherapy, PTT, PDT, and PIT over the fiscal years from 2000 to 2023. In PTT, funding has generally increased over the years, with fluctuations in certain years. There was a noticeable increase in funding from 2019 onward, reaching its peak in 2022. In PDT, funding shows variations over the years, with some fluctuations but a relatively stable trend. It peaked in 2003 and 2004, followed by a decrease in subsequent years. In immunotherapy, funding has shown a steady increase over the years, with significant growth observed, particularly from 2010 onward. The highest funding allocation for immunotherapy was observed in recent years, with a substantial increase from 2017 to 2022. In PIT, funding has also shown fluctuations over the years, with some years experiencing increases while others showing decreases. Funding for PIT has generally increased over time, with a notable peak in 2018, followed by fluctuations in subsequent years. Beyond NIH funding, other global agencies also support these fields. The Canadian Institutes of Health Research has allocated CAD 252.5 million to immunotherapy, while PIT funding remains significantly lower at CAD 3.6 million, compared to PDT (CAD 7.8 million) and PTT (CAD 4.4 million). UK Research and Innovation has invested £86.7 million in immunotherapy, with lower funding for PDT (£13.6 million) and PTT (£1.5 million). Similarly, the European Research Council has provided €174 million for immunotherapy, while PDT and PTT receive significantly less (€2 million and €150000, respectively).

Vaccines have secured the highest funding, amounting to $3,053,172,719, reflecting substantial investment, as shown in Figure 8E. Checkpoint inhibitors follow closely behind with $565,521,994 in funding, highlighting their significance as well. ACT therapies have also garnered substantial support, totaling $1,042,188,713, emphasizing the growing interest in personalized treatments harnessing the patient's immune cells. Additionally, cytokines and oncolytic viruses have also received significant funding. Despite variations in funding levels, these modalities collectively demonstrate the diverse approaches and ongoing efforts to advance immunotherapy research and development for cancer treatment.

It is also worth considering the funding landscape of clinically viable nanoparticles and nanomaterials, Figure 8F. Notably, exosomes have received significant funding, totaling $22,419,495, reflecting a growing interest in harnessing these naturally occurring vesicles for targeted drug delivery and diagnostics. Silica and gold nanoparticles also garnered substantial funding, amounting to $16,217,362 and $9,945,756, respectively, underscoring their versatility and potential in therapeutic and imaging applications. Polymeric nanoparticles and dendrimers have also secured notable funding, suggesting ongoing research into their development as drug‐delivery systems. However, carbon nanotubes and graphene received comparatively lower funding, indicating potential areas for further exploration and investment in nanomaterial research. Overall, these data suggest a growing interest and investment in immunotherapy and PIT, particularly in recent years, reflecting its potential as a promising treatment modality for cancer. Additionally, while PTT and PDT have seen fluctuations in funding, they continue to receive significant support, indicating ongoing research and development efforts in these areas.

On the other hand, the calculation of the extent of investment in nanomedicine‐assisted phototherapies in the private sector is challenging because it involves evaluating not just direct financial outlays but also navigating proprietary and competitive factors, such as undisclosed R&D budgets, partnership deals, and intellectual property costs. However, despite the fact that nanomedicines and phototherapies face strong criticism and several terminations on the approval road, a few leading companies like Rakuten and Immunophotonics are heading toward putting PINs into the market.

Rakuten Medical, Inc., formerly known as Aspyrian Therapeutics, is a privately funded, global biotechnology, clinical‐stage company driven by its strategic focus on precision investigational therapies developed on the Illuminox platform.^[^ ^135^ ^]^ This platform, rooted in PIT, displays the potential to selectively target and induce cell death and tumor necrosis through the use of non‐thermal light activation. ASP‐1929, a pivotal antibody‐drug conjugate within Rakuten Medical's pipeline, shows promise in its capacity to bind with EGFR prevalent in various solid tumors.^[^ ^136^ ^]^ Upon activation by NIR light, ASP‐1929 triggers a biophysical cascade that ultimately leads to cell death and degradation of tumor tissue.^[^ ^137^ ^]^ With conditional early approval from the Japanese Ministry of Health, Labor, and Welfare, ASP‐1929 is in the midst of a global phase III clinical trial for recurrent head and neck cancer. Rakuten Medical is taking a comprehensive approach, advancing not only the ASP‐1929 monotherapy trial but also investigating combinatorial therapy with other drugs and checkpoint inhibitors. Additionally, Rakuten Medical is investigating another molecule, RM‐1995, in an early‐stage clinical trial (jRCT number: 2031220721). It has reached Phase I for the locally advanced head and neck squamous cell carcinoma and cutaneous squamous cell carcinoma indications.

Rakuten Medical has raised up to $500 million, and in 2019, it also secured an additional $100 million in Series C‐1 Preferred Stock financing on July 31, 2019, from Rakuten, Inc., a prominent Japanese internet services leader. This funding strengthened the expansion of PIT platform development, enhanced business functions, and explored new investigational compounds for treating various cancers. Further, Hikma Pharmaceuticals, a public limited company, has entered into an exclusive licensing and commercialization agreement with Rakuten Medical, Inc.^[^ ^138^ ^]^ This multinational pharmaceutical company is partnering with Rakuten Medical. According to the terms, Hikma gains an exclusive license to market Rakuten Medical's pipeline products using the Alluminox (formerly known as Illuminox) PIT platform in its Middle East and North Africa markets.^[^ ^139^ ^]^

Immunophotonics is another clinical‐stage immunoncology company that treats solid tumors.^[^ ^140^ ^]^ It's headquartered in Missouri, USA, with subsidiaries in different countries like China and Switzerland. IP‐001 is the product that potentially transforms solid tumor ablation into active immunotherapy for cancer.^[^ ^141^ ^]^ The patents related to this technology have been protected in more than 40 countries. It has received around $30.4 M from 13 investors, and the latest being are Zubizoom Investments and iSelect Fund. Recently, Immunophotonics raised $3.5 M from Venture Round.^[^ ^142^ ^]^

In summary, the growing investments in PIT and PINs highlight their increasing impact on cancer therapy. While government funding trends show steady support, the private sector, led by companies like Rakuten Medical and Immunophotonics, is actively driving commercialization efforts despite regulatory hurdles. These advancements reinforce the potential of nanomedicine‐based immunotherapies in shaping future treatment strategies.

## 5 Challenges for the Translation of PINs

### 5.1 Interactions of PINs with Biological Components

The interactions between PINs and biological components in terms of toxicity present a complex landscape. Various factors can contribute to potential toxic effects that need to be considered. The immediate phototoxicity is a concern when PINs are activated by light, potentially producing ROS or localized heat.^[^ ^143^ ^]^ While this can be beneficial for targeted treatments, it could inadvertently harm surrounding healthy cells and tissues if not properly managed.^[^ ^144^ ^]^ The ROS overproduction can lead to oxidative stress and cellular damage. The nanomaterial toxicity may arise from the inherent properties of the base materials used in nanomedicine.^[^ ^145^ ^]^ Certain metals, metal oxides, or organic polymers might carry cytotoxic effects due to ROS generation, interference with cellular processes, or other unexpected mechanisms.^[^ ^146^ ^]^

The accumulation of PINs in unintended sites can also lead to unintended phototoxicity or drug effects in healthy tissues. While contributing to desired immunomodulation, immunogenicity could lead to uncontrolled or excessive immune responses. The body might perceive PINs as foreign bodies, resulting in inflammation, tissue damage, or other complications.^[^ ^147^ ^]^ Also, long‐term accumulation concerns can arise if PINs are not efficiently cleared from the body. This can lead to potential accumulation in organs like the liver and/or spleen, and prolonged exposure might trigger organ dysfunction or damage.^[^ ^148^ ^]^ A major safety concern is systemic toxicity, where broad distribution in the bloodstream can lead to off‐target phototoxic effects in non‐tumor tissues, posing risks of tissue damage upon light activation. Furthermore, the possibility of inducing a tolerogenic T cell response—where the immune system becomes desensitized to tumor antigens carried by PINs—could weaken immune surveillance and reduce therapeutic efficacy. The surface coatings and additives applied to PINs to enhance stability, biocompatibility, or targeting can also introduce toxicity concerns.^[^ ^149^ ^]^ The interaction with other medications introduces the possibility of PINs interacting with concurrently administered drugs or therapies, potentially causing unforeseen toxic effects or diminishing the effectiveness of other treatments. To mitigate these potential toxicities, researchers need to undertake comprehensive in vitro and in vivo studies during the development of PINs. The balance of therapeutic benefits with potential risks is crucial, involving optimization of design and dosage to ensure the safety of these novel treatments.

### 5.2 Technical Challenges in Production and Quality Assurance

The development of PINs is a multifaceted endeavor with several technical challenges. The synthesis and scalability of these PINs are complicated, particularly when combining components such as gold nanorods/nanoshells for photothermal properties with immunomodulatory drugs within polymeric nanoparticles, liposomes, or other nanocarriers. The assurance of uniform size and shape, vital for nanoparticle behavior in the body, is compounded by the need to scale up production for larger quantities.

The limited penetration of NIR light restricts the ability to activate PINs in deeper tissues, which is essential for many clinical applications. To address this, fiber‐optic or endoscopic light delivery systems have emerged as promising solutions, allowing clinicians to deliver light precisely to target tissues deep within the body. These devices can be positioned close to the tumor through minimally invasive procedures, maximizing light exposure at the desired location while minimizing damage to surrounding healthy tissue. Clinical trials are underway exploring these delivery systems, especially in gastrointestinal, urological, and pulmonary cancers, where endoscopically guided light delivery can provide targeted activation of phototherapies. For example, ongoing trial‐ NCT04240639 is evaluating the safety and efficacy of fiber‐optic NIR systems for advanced prostate and bladder cancers, achieving promising localized therapeutic effects. As trials progress, these methods hold potential to expand phototherapy applications across challenging, deep‐seated tumors.

The balance of biocompatibility and potential toxicity presents another hurdle, particularly with materials beyond gold nanoparticles. The by‐products after light activation or nanoparticle degradation could pose toxicity risks, and the breakage of photo‐responsive polymers by NIR light might yield toxic products.^[^ ^150^ ^]^ The management of the controlled release of immunomodulators in response to light demands a deep understanding of photothermal and drug delivery complexities. The striking of the right balance is essential; for example, releasing immunomodulators too quickly upon NIR light exposure could lead to system flooding and severe side effects, while releasing them too slowly might undermine therapeutic efficacy.

The stability, storage, and in vivo behavior also challenge PINs translation. The unintended changes or degradation over time could impact performance, with light‐sensitive linkers becoming less responsive due to improper storage or unintended light exposure. Moreover, the complicated production processes and advanced materials drive up costs, as seen in the clinical trials of light‐responsive nanoshells such as Auroshell (NCT01679470), which require clinical‐grade materials, specialized equipment, and GMP facilities, ultimately delaying its translation.

### 5.3 Controversial EPR

The EPR effect is the gold standard for passive drug delivery and is very useful for the nanomedicine‐driven PTT, PDT, and PIT.^[^ ^151^ ^]^ However, as more research has been conducted in this direction, it is becoming controversial, with claims that “EPR fails in clinical trials” or it only works in rodents, not humans. It is worth noting that EPR is very variable and shows significant variation between patients and different tumor types, or even the same tumor at different times. A meta‐analysis of nanomedicine studies from the past 10 years found that only 0.7% of the injected dosage was delivered to the tumor.^[^ ^152^ ^]^

### 5.4 Poor Pharmacokinetics

Most of the administered nanoparticles are removed by the mononuclear phagocyte system (MPS) in a matter of time.^[^ ^153^ ^]^ Similarly, this poses the risk of non‐specific distribution of PINs to healthy organs and affects their efficacy. To address this, surface functionalization of PINs is in trend. For example, PINs are coated with PEG or other biomimetic membranes to prevent the binding of plasma proteins, reducing MPS clearance and increasing circulation time. However, this approach has been associated with challenges such as immunogenicity, toxicity, and inadequate internalization of PINs.^[^ ^154^ ^]^ This has led to the functionalization with a more biological‐like biomimetic membrane and “do not eat me” peptides to camouflage nanoparticles.^[^ ^4g^ ^]^

### 5.5 Limited Reliability and Validity of Animal Models

The unavailability of precise animal models for recapitulating the human malignancy is a well‐noted issue in nanomedicines‐driven immunotherapy, PTT, PDT, and PIT.^[^ ^155^ ^]^ This has been a primary reason for the discrepancies between preclinical and clinical outcomes. For example, most preclinical studies on photo therapies are conducted on immune‐deficient animals, which do not allow for the prediction of adverse effects in clinical trials.^[^ ^156^ ^]^ Additionally, there is a huge difference between tumor sizes in animals and that develops naturally in humans.

### 5.6 Other Potential Reasons for Clinical Slow Progress

Apart from the design, there are other factors also that contribute to the slow progress or even failure of immunotherapy and phototherapies clinical trials. These include the immunomodulator not being effective for the given indication, improper selection of patients in clinical trials, inappropriate combinations of immunomodulators with photo‐responsive/activable agents, as well as regulatory concerns.

### 5.7 Bridging the Gap

The successful clinical translation of PINs requires interdisciplinary collaboration and technological integration across nanomedicine, immunology, bioengineering, and clinical sciences. The development of safer and more effective PINs relies on coordinated efforts between material scientists optimizing nanoparticle formulations, immunologists ensuring biocompatibility and immune modulation, and engineers advancing light‐delivery technologies for deep‐tissue activation.

To overcome manufacturing and scalability challenges, bioengineers and material scientists must refine GMP‐compliant production methods, nanoparticle stability, and batch reproducibility.^[^ ^157^ ^]^ Given the variability of the EPR effect and limitations of current preclinical models, integrating computational modeling and organoid‐based systems can help predict PIN biodistribution and therapeutic responses, bridging the gap between preclinical studies and human trials.^[^ ^158^ ^]^ Moreover, interdisciplinary collaboration among oncologists, regulatory scientists, and industry stakeholders is necessary to develop standardized treatment protocols, safety guidelines, and regulatory pathways for the efficient clinical adoption of PINs.

In summary, the translation of PINs is hindered by toxicity risks, scalability issues, poor pharmacokinetics, limited light penetration, and interdisciplinary collaboration. Challenges such as EPR variability, regulatory hurdles, and inadequate animal models further impact clinical progress. Overcoming these barriers requires advancing targeted delivery, improving safety assessments, and refining clinical trial designs. By fostering interdisciplinary integration, aligning technological innovations with clinical needs, and establishing standardized evaluation criteria, PIN therapies can be optimized for enhanced efficacy, safety, and widespread clinical application.

## 6 Future Direction and Conclusion

The future of PINs holds immense promise, particularly in advancing personalized immunotherapy for cancer and other immune‐related disorders. These cutting‐edge nanomedicines offer the potential to incorporate patient‐specific antigens or immunomodulators, enabling tailored treatments that address the unique immunological landscape of each individual. This level of customization could transform the precision of cancer care, paving the way for therapies that are not only more effective but also minimize off‐target effects and associated toxicities. From a clinical perspective, the integration of PINs with existing treatment modalities such as chemotherapy and radiotherapy offers exciting possibilities for synergistic effects. For example, PINs could enhance the immunogenicity of tumors treated with radiotherapy or amplify the effects of immune checkpoint inhibitors by strategically modulating immune pathways. Emerging research supports the hypothesis that these nanomedicines, when combined with conventional therapies, may overcome resistance mechanisms that often limit treatment success. Further innovations in combination strategies could unlock greater therapeutic potential, offering new hope for patients with advanced or refractory cancers.

One of the most transformative prospects of PINs lies in their integration with nanosensors for real‐time monitoring of the immune response. Clinicians could leverage these systems to dynamically adjust treatments based on patient‐specific immune activity. This capability would allow for precise modulation of immune pathways, such as activating cytotoxic T cells in the tumor microenvironment or suppressing regulatory T cells that hinder anti‐tumor responses. The photo‐responsive/activable nature of PINs adds another layer of control, enabling spatially and temporally targeted interventions that minimize collateral damage to healthy tissues. Despite these promising prospects, the journey toward clinical adoption of PINs requires addressing significant challenges. Rigorous clinical trials are imperative to establish their safety, efficacy, and biocompatibility across diverse patient populations. Standardization in the design, manufacturing, and characterization of PINs is essential to ensure reproducibility and regulatory approval. Additionally, understanding the long‐term effects of these nanomedicines on immune homeostasis and potential off‐target immune reactions remains a critical area of investigation.

For clinicians, the integration of PINs into therapeutic regimens will necessitate new protocols and training to harness their full potential. Interdisciplinary collaboration among oncologists, immunologists, and nanotechnology experts will be pivotal in translating laboratory findings into practical, life‐changing treatments. While challenges persist, the promise of PINs as a revolutionary tool in personalized medicine is undeniable, holding the potential to reshape the landscape of immunotherapy and redefine clinical outcomes for patients worldwide.

## Conflict of Interest

The authors declare no conflict of interest.

## Author Contributions

D.S.C. gathered data for the article, wrote the first draft and prepared figures and tables under the supervision and contributions of C.S.U. D.S.C., G.H., and C.S.U. contributed to the conceptualization, revision, and finalization of the review. K.S., R.H.N., D.J.Y., and M.S. were involved significantly in discussions about the content, as well as the writing, reviewing, and editing of the manuscript before submission.
